# Microfluidics-enabled fluorinated assembly of EGCG-ligands-siTOX nanoparticles for synergetic tumor cells and exhausted t cells regulation in cancer immunotherapy

**DOI:** 10.1186/s12951-024-02328-4

**Published:** 2024-03-04

**Authors:** Xiaowei Han, Guozheng Zhang, Xiaozhen Wu, Shufeng Xu, Jiahuan Liu, Kaikai Wang, Tianqing Liu, Pengkai Wu

**Affiliations:** 1 Department of Radiology, The Quzhou Affiliated Hospital of Wenzhou Medical University, Quzhou People’s Hospital, Quzhou, China; 2 Department of Hepatobiliary Surgery, Innovative Institute of Tumor Immunity and Medicine (ITIM), Anhui Province Key Laboratory of Tumor Immune Microenvironment and Immunotherapy, The First Affiliated Hospital of Anhui Medical University, Hefei, 230022 Anhui China; 3 School of Pharmacy, Nantong University, Nantong, 226001 China; 4 NICM Health Research Institute, Western Sydney University, Sydney, NSW 2145 Australia

**Keywords:** EGCG, Fluorine, siRNA delivery, Exhaustion, Immunotherapy

## Abstract

Immune checkpoint inhibitor (ICI)-derived evolution offers a versatile means of developing novel immunotherapies that targets programmed death-ligand 1 (PD-L1)/programmed death-1 (PD-1) axis. However, one major challenge is T cell exhaustion, which contributes to low response rates in "cold" tumors. Herein, we introduce a fluorinated assembly system of LFNPs/siTOX complexes consisting of fluorinated EGCG (FEGCG), fluorinated aminolauric acid (LA), and fluorinated polyethylene glycol (PEG) to efficiently deliver small interfering RNA anti-TOX (thymus high mobility group box protein, TOX) for synergistic tumor cells and exhausted T cells regulation. Using a microfluidic approach, a library of LFNPs/siTOX complexes were prepared by altering the placement of the hydrophobe (LA), the surface PEGylation density, and the siTOX ratio. Among the different formulations tested, the lead formulation, LFNPs3-3/siTOX complexes, demonstrated enhanced siRNA complexation, sensitive drug release, improved stability and delivery efficacy, and acceptable biosafety. Upon administration by the intravenous injection, this formulation was able to evoke a robust immune response by inhibiting PD-L1 expression and mitigating T cell exhaustion. Overall, this study provides valuable insights into the fluorinated assembly and concomitant optimization of the EGCG-based delivery system. Furthermore, it offers a promising strategy for cancer immunotherapy, highlighting its potential in improving response rates in ‘‘cold’’ tumors.

**Graphical Abstract:**

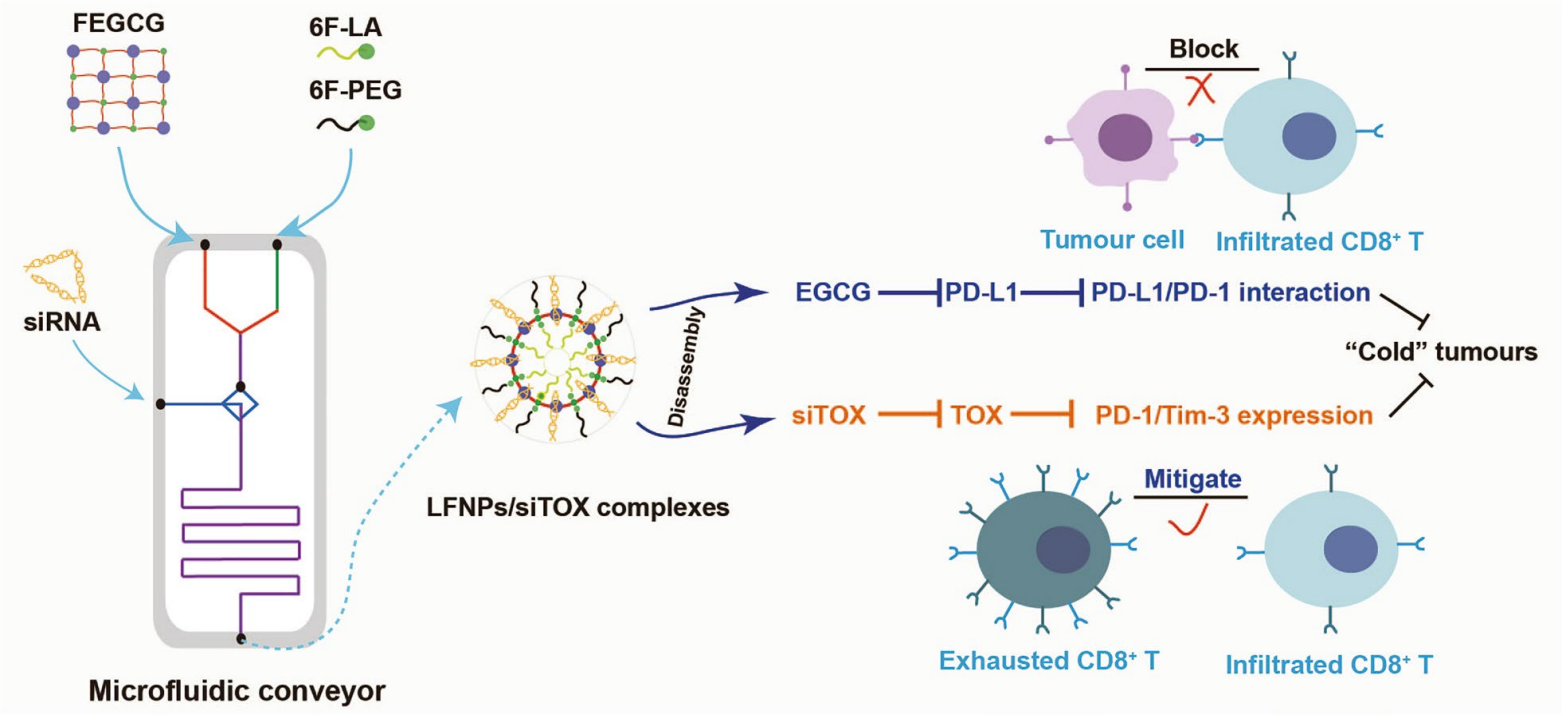

**Supplementary Information:**

The online version contains supplementary material available at 10.1186/s12951-024-02328-4.

## Introduction

Cancer is the leading cause of death globally, resulting in significant medical challenges. Among the various approaches to treatment, immunotherapy using immune checkpoint inhibitors (ICIs) has shown promise in activating the immune system of patients [1, 2]. The interference of the programmed death-ligand 1 (PD-L1)/programmed death-1 (PD-1) axis, as a representative pathway of ICI therapy, has been extensively researched to prevent tumor cell immune escape and enhance immune response [3]. Notably, antibody drugs targeting PD-L1/PD-1 have been approved by the U.S. Food & Drug Administration for the treatment of various malignancies [4]. Despite the significant clinical benefits of PD-L1/PD-1 blockade, a low response rate is still observed in patients with "cold" tumors, indicating limited CD8^+^ T cell infiltration [5, 6]. T cell exhaustion, characterized by the limited or lost function and proliferation of T cells, poses a major obstacle to effective CD8^+^ T cell-mediated tumor cell killing [7, 8]. Therefore, combining PD-L1/PD-1 blockade with strategies to mitigate T cell exhaustion holds promise for enhancing the immune response in cancer immunotherapy.

In pursuit of this goal, extensive research has been conducted to understand the differentiation and function of exhausted T cells and identify potential targets for successful immunotherapy. In cases of acute infections, T cells exhibit a classical differentiation pattern involving antigen clearance through expansion, contraction, and memory phases. However, T cells responding to persistent infections or chronic antigens experience a dysfunctional state characterized by reduced secretion of cytotoxic cytokines and increased expression of inhibitory receptors [9, 10]. Despite their impaired function, exhausted T cells retain some ability to control tumor growth, making their mitigation a viable approach. In recent years, the importance of thymocyte selection-associated high mobility group box (TOX) as a key regulator in the field of exhausted T cells has been demonstrated. In addition to being essential for the definitive identification and epigenetic remodeling of exhausted T cells, TOX also plays a critical role in their differentiation and development. Without TOX, the formation of exhausted T cells is largely impaired. Interestingly, the function of TOX in T cell differentiation is specific to exhausted T cells, as it is largely dispensable for the formation of effector and memory CD8^+^ T cells. This indicates that TOX plays a unique and crucial role in the context of T cell exhaustion [11–16]. Encouragingly, studies have shown that mitigating T cell exhaustion and enhancing cytotoxic molecules can be achieved by deleting or knocking down TOX [8, 17]. Therefore, interfering with TOX using small interfering RNA (siRNA) presents a feasible approach for mitigating T cell exhaustion, as supported by the findings of our previous study [8].

The critical challenge in the application of siTOX delivery lies in the selection of a suitable platform that takes into account the in vivo electronegativity and instability of siRNA [18, 19]. Epigallocatechin gallate (EGCG), a prominent constituent found in green tea, has garnered significant attention in both laboratory and clinical investigations due to its diverse range of bioactivities. These encompass anti-oxidant, anti-inflammatory, anti-viral, and anti-cancer effects. Notably, EGCG has been classified as 'Generally Recognized as Safe' (GRAS) and exhibits promising potential as a PD-L1 inhibitor to modulate the immune response in tumor therapy. Furthermore, the ample presence of hydroxyl and benzene rings in EGCG contribute to its robust binding affinity with various guest molecules, such as siRNA, proteins, and chemical drugs [20–22]. In our previous study, we utilized fluorination as an effective strategy to enhance siRNA delivery of EGCG by exploiting the lipophobic and bioinert properties of fluorocarbons [23]. In this study, we aimed to further improve the performance of EGCG polyplexes by employing a combination of optimized polyethylene glycol (PEG) and hydrophobic components. PEGylation is a common strategy to prevent the clearance of polyplexes from the mononuclear phagocyte system through electrostatic interaction [24, 25]. Additionally, the incorporation of hydrophobicity into the polyplexes serves as another strategy to enhance siRNA stability and delivery through hydrophobic interaction [26, 27]. Hence, the integration of fluorination, PEGylation, and hydrophobic components in EGCG polyplexes resulted in the development of a highly effective and safe EGCG-based system for siRNA delivery in vivo.

**Fig. 1:**
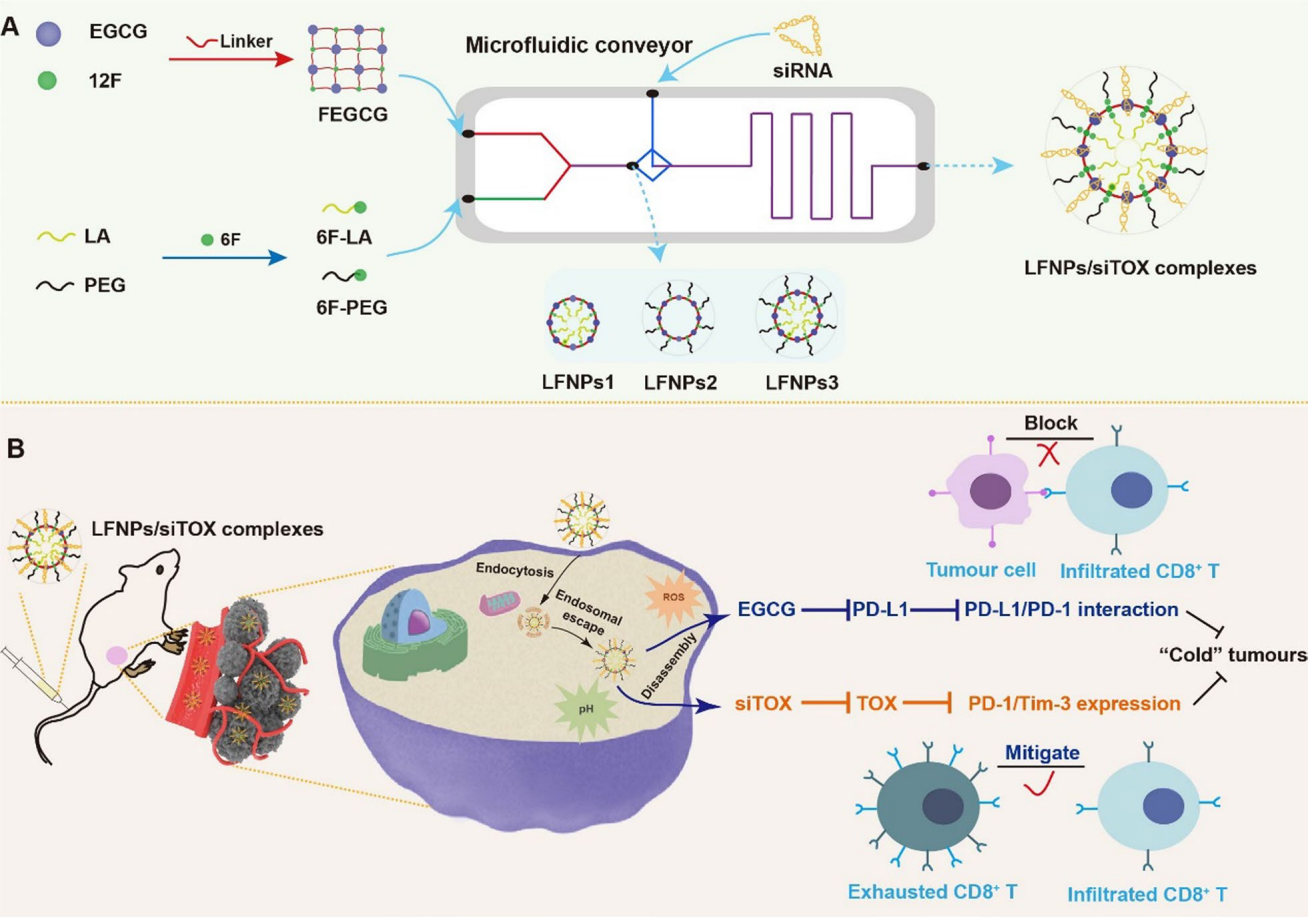
Preparation and regulatory mechanism of LFNPs/siTOX complexes. **A** Preparation of LFNPs/siTOX complexes through a microfluidic device. FEGCG, 6F-LA, and 6F-PEG were first synthesized and reacted to construct LFNPs via fluorine interaction. LFNPs/siTOX complexes were next formed with siTOX via hydrogen-bond interaction. **B** Transition from “cold” to “hot” tumors depends on the synergetic regulation of tumor cells and exhausted T cells by LFNPs/siTOX complexes. LFNPs can inhibit PD-L1 expression to block the PD-1/PD-L1 interaction. siTOX can decrease TOX expression to mitigate T cells exhaustion. Their combination can increase the infiltration of CD8 + T cells to fight “cold” tumors

Herein, we synthesized fluorinated ECGC (FEGCG) with reactive oxygen species (ROS) sensitive profile, as well as fluorinated aminolauric acid (LA) and PEG (6F-LA and 6F-PEG) with pH sensitive profile via polymerization and Schiff base reaction. Ligand-functionalized EGCG-based nanoparticles (LFNPs) were then formed through self-assembly driven by fluorine interaction, with variations in the components of the LA core (LFNPs1), PEG corona (LFNPs2), and both the LA core and PEG corona (LFNPs3). LFNPs/siTOX complexes were prepared using LFNPs and siTOX through hydrogen-bond interaction. To achieve rapid synthesis and screening of the lead formulation, we employed a microfluidic approach as an advanced technology for the production, testing, and application of NPs, taking advantages of its ability to simulate dynamic fluid flows, gradients, and specific microenvironments. As a result, we were able to fabricate a series of LFNPs1, LFNPs2, LFNPs3, and LFNPs/siTOX complexes with high reproducibility, simple operation, and time effectiveness, in comparison to conventional methods such as the dropwise method [28–30]. Therefore, microfluidics is highly suitable for ensuring the controlled synthesis of NPs with consistent physicochemical properties, ultimately leading to the desired therapeutic efficacy in vivo (Fig. 1A). In this study, the lead formulation of LFNPs3-3/siTOX complexes exhibit superior properties such as enhanced siRNA complexation, ROS/pH-triggered drug release, improved stability and delivery efficacy, as well as acceptable biosafety. Upon systemic administration, LFNPs/siTOX complexes not only inhibit PD-L1 expression to impede the PD-1/PD-L1 interaction but also reduce TOX expression to mitigate T cell exhaustion. The synergistic regulation of tumor cells and exhausted T cells can promote the infiltration of CD8^+^ T cells, thus fighting against "cold" tumors (Fig. 1B). This combinatorial approach provides a comprehensive exploration of the structure–function relationship in the field of EGCG-based systems and holds promise as a strategy for cancer immunotherapy.

## Materials and methods

### Synthesis of FEGCG, 6F-LA and 6F-PEG

The fluoropolymers of FEGCG were synthesized by one-step polymerization, and detailed steps were shown in our previous study [23]. 6F-Ben modified LA or PEG was synthesized by the Schiff base reaction between the amino groups (–NH_2_) of LA/PEG and the aldehyde groups (–CHO) of 6F-Ben under the catalysis of trace acetic acid for 24 h. Briefly, LA (215.3 mg, 1 mM)/PEG (5000 mg, 1 mM) and 6F-ben (242.1 mg, 1 mM) were co-dissolved in 5 mL dry CH_3_OH with slight trace acetic acid, and the reaction was kept at room temperature for 24 h. The column chromatography was used to purify 6F-LA (CH_2_Cl_2_:CH_3_OH, 10:1), and dialysis/lyophilization method was used to purify 6F-PEG (molecular weight cut off 3.5 kDa). The structure of obtained 6F-LA and 6F-PEG was characterized by ^1^H-NMR and ^19^F-NMR (Bruker 600 MHz).

### Preparation and characterization of LFNPs and LFNPs/siRNA complexes by Microfluidics

A market microfluidic device (Dolomite, U.K.) was used to prepare LFNPs and LFNPs/siRNA complexes. Unless otherwise indicated, the composition of FEGCG/LFNPs was expressed as equivalent EGCG (i.e., excluding the F, LA and PEG content). For LFNPs generation, FEGCG solution with a concentration of 1 mg/mL were injected into one channel, and 6F-LA/6F-PEG solution was injected into another channel at corresponding w/w ratio (i.e., 5% 6F-LA = 0.05 mg/mL). For LFNPs/siRNA complexes generation, siRNA solution with a concentration of 0.04 mg/mL were injected into the first channel, and LFNPs solution of 1 mg/mL were injected into another channel at w/w 25. The resultant products were collected at the outlet and stored at 4  C for further use. Their particle size, polydiseperse index (PDI) and zeta potential were determined using dynamic light scattering (DLS) (Malvern Instruments Ltd., U.K.). The particle morphology was carried out using transmission electron microscope (TEM, JEM1200EX, 100 kV).

### Preparation of LFNPs/siRNA complexes by manual mixing

The FEGCG/LFNPs and siRNA concentrations were kept the same as that of used in microfluidic preparation. The complexes were prepared by vigorous pipetting of siRNA solution with FEGCG/LFNPs to achieve the desired complexes and incubated for 30 min before use.

### EB competitive binding assay

The interaction of EGCG, FEGCG and LFNPs3 sub class with siRNA was characterized using an EB competitive assay. Generally, the above complexes prepared at different w/w ratios were incubated with EB solution at a w/w ratio of 10:1 (siRNA/EB), and then the mixed solution was kept for further 10 min to measure the changed fluorescence intensity of EB (excitation at 470 nm, emission at 520 nm). EB solution and EB/siRNA complexes were served as negative and positive controls, respectively. The siRNA condensation efficiency (%) reflected in the changed EB fluorescence intensity was defined as:

$${\text{Relative EB fluorescence intensity }}\left( {\text{\% }} \right) = {{F_{EB/siRNA} - F_{treatments} } \mathord{\left/ {\vphantom {{F_{EB/siRNA} - F_{treatments} } {F_{EB/siRNA} - F_{EB} }}} \right. \kern-0pt} {F_{EB/siRNA} - F_{EB} }} \times 100$$

### Fluorescence quenching assay and agarose gel electrophoresis

The quenching of EGCG, FEGCG and LFNPs3 sub class with Cy5-siRNA was characterized using a fluorescence quenching assay. Briefly, the above complexes prepared at w/w 25 (siRNA concentrations = 266 μg/mL) were incubated for 30 min and measured for the fluorescence scanning spectrum (excitation at 560 nm, emission at 620–740 nm for Cy5-siRNA).

The siRNA condense ability of above solutions was determined by agarose gel electrophoresis at 100 V for 15 min with complexes prepared at w/w 25 (siRNA concentrations = 40 μg/mL), and the encapsulation efficiency was quantified by ImageJ.

### ROS/pH triggered disassembly and stability of LFNPs3-3 complexes

ROS/pH triggered siRNA release of LFNPs3-3 complexes was evaluated by determining the changed Cy5 fluorescence intensity, hydrodynamic size, and TEM morphology by incubation them in pH 7.4 PBS, pH 5.0 PBS, H_2_O_2_ with pH 7.4 PBS, and H_2_O_2_ with pH 5.0 PBS, respectively.

The stability of LFNPs3-3 complexes was performed by incubating them with PBS (pH 7.4) with or without FBS for 48 h. The hydrodynamic size change was measured by DLS to reflect their stability. The resistance of different complexes to heparin sulfate was compared by FRET assay. Briefly, EGCG/FEGCG/LFNPs3 sub class complexes prepared with Cy3-siRNA and Cy5-siRNA at w/w ratio of 25:1:1 (266 μg/mL siRNA) were incubated with heparin (20 U/mL) for 12 h, and then performed by multimode reader for FRET analysis.

### Cytotoxicity, cellular uptake and endo/lysosomes escape

The cytotoxicity of EGCG, FEGCG and LFNPs3 sub class were measured by CCK8 and LDH assay in 4T1 cells and exhausted T cells, respectively. Briefly, cells were seeded in 96-well plates until ~ 80% density. The treatments with different concentrations were incubated with cells for 24 h. After that, a standard CCK8 or LDH assay was performed to determine the cytotoxicity.

Cellular uptake and endo/lysosomes escape of the complexes prepared with FAM-siRNA (100 nM) at w/w 10 was analyzed after incubation them with 4T1 cells or T cells for 4 h. After that, the fluorescence intensity in cells were detected by flow cytometry. Cellular uptake and Endo/lysosomes escape was further assessed by confocal microscopy.

### Luc gene silencing

Luc gene silencing in vitro was performed using 4T1.Luc cells that seeded in 48-well plates. The complexes prepared with siLuc (100 nM, w/w 10) were incubated with the above cells at the condition without or with 30%/50% FBS. After 4 h, the formulations were removed, and the cells were re-incubated with fresh medium for another 24 h. The final cells were washed and lysed to detect the Luc activity according to the protocol of luciferase assay kit (Beyotime, China).

Luc gene silencing in vivo was performed using 4T1.Luc tumor model. The tumor model was put into use until the tumor grown to ∼100 mm^3^. All animals were divided into 5 groups (n = 3) and administered with related complexes (siScr/siLuc, 1 mg/Kg siRNA, w/w 25) by i.v. injection. The total flux of the region of interest (ROI) was analyzed while ensuring consistency in all measurement parameters on day 0 and day 2. After imaging, the tumors were excised and homogenized to further measure the Luc expression using luciferase assay kit.

### PD-L1 expression, TOX expression, and the state of exhausted T-cells in vitro

The PD-L1 silencing ability was evaluated by incubating 4T1 cells with IFN-γ (10 ng/mL) for 12 h, and further treating them with FEGCG/siScr, LFNPs3-3/siScr, LFNPs3-3/siTOX (100 nM, w/w 10), and α-PD-L1 (10 µg/mL) for 24 h. The TOX silencing ability was evaluated by incubating exhausted CD8^+^ T cells with FEGCG/siScr, LFNPs3-3/siScr, LFNPs3-3/siTOX, Lipo3000/siScr, and Lipo3000/siTOX for 24 h. The expression of PD-L1 and TOX were determined by flow cytometry and western blot. In addition, exhausted CD8^+^ T cells isolated from tumor tissues or simulated from non-exhausted T cells were incubated with the treatments of FEGCG/siScr, LFNPs3-3/siScr, and LFNPs3-3/siTOX to count the percentage of PD-1^+^Tim-3^+^ cells.

### Biodistribution and biosafety study

For biodistribution study, female Balb/c mice were randomly divided into 2 groups of FEGCG complexes and LFNPs3-3 complexes (n = 3). At 6 h and 12 h after Cy5-siRNA complexes (1 mg/Kg siRNA) administration by the intravenous injection (i.v.), the animals were sacrificed to excise major organs including heart, liver, spleen lungs, and kidneys. These organs were imaged, and the average radiance of ROI without background was quantified using Caliper IVIS image system (excitation at 630 nm, emission at 680 nm).

For test the potential toxicity, female Balb/c mice were randomly divided into 3 groups of saline, FEGCG and LFNPs3-3 complexes (n = 3). After 12 h of i.v. injection, animals were sacrificed and the major organs (heart, liver, spleen lungs, and kidneys) and blood were collected for biochemical analysis. H&E staining was performed to determine the histopathological changes in organs. Alanine aminotransferase (ALT) and aspartate aminotransferase (AST) levels were determined to reflect liver functions. Blood urea nitrogen (BUN) and creatinine (CREA) levels were determined to reflect kidney functions. The hemolysis assay of FEGCG/siScr, LFNPs3-3/siScr, LFNPs3-3/siTOX, and Triton were evaluated by incubating them with isolated mouse red blood cells at 37 ℃ for 4 h. Afterwards, the sample was centrifuged for analysis, and then the absorbance of collected supernatant was measured at 540 nm.

### Therapeutic efficacy and immune activation mechanism in vivo

For *in vivo* antitumor and antimetastatic studies, the mice were randomly divided into four groups of saline, FEGCG/siScr, LFNPs3-3/siScr and LFNPs3-3/siTOX when the tumor size reached ~ 150 mm^3^. In some cases, FEGCG/siTox group was added to compare its anti-tumor efficacy with LFNPs3-3/siTOX treatment. The different treatments were performed on day 0, day 2, day 4, day 6, day 8, and day 10 for total six times (1 mg/Kg siRNA, w/w 25) by i.v. injection. The tumor weight and body weight were monitored on day 19, and the survival analysis was monitored until day 60. Lungs were harvested to count the metastatic nodules and H and E staining.

To investigate the immune activation mechanism, PD-L1 expression in isolated tumor cells was examined. The collected lymphocytes were then stained with anti-CD45, anti-CD3, and anti-CD8 antibodies, and analyzed by flow cytometry to determine the number of CD8^+^ T cells. Exhausted CD8^+^ T cells were purified and stained with anti-CD8, anti-PD-1, anti-Tim3, anti-TOX, anti-IFN-γ, and anti-Granzyme B antibodies, following a standard surface/nuclear marker staining protocol, to determine the expression of TOX, IFN-γ, Granzyme B, and PD-1^+^/Tim3^+^. Levels of TNF-α and IFN-γ in blood samples were measured using ELISA.

### Statistical analysis

Statistical analysis was performed by an unpaired two-tailed Student’s t-test for two groups and one-way analysis of variance (ANOVA) with Turkey’s multiple comparisons for multiple groups (when *P < 0.05, **P < 0.01, ***P < 0.001, ****P < 0.0001, the data was considered statistically significant).

## Results

### Synthesis and characterization of LFNPs and LFNPs/siRNA complexes

Surface chemistry is an attractive approach to change the physicochemical properties of NPs in vitro and in vivo. Usually, a library of modified NPs needs to be prepared to explore the effects of different ligands on their performance [31, 32]. However, it is usually not easy to obtain a large number of NPs due to the complex and uncontrollable synthetic process. Therefore, microfluidic technology can be used as a feasible approach to prepare and screen different LFNPs for siRNA delivery in this study. Thus, FEGCG was synthesized by crosslinking EGCG with fluorine atoms as the bridge through a ROS sensitive linker. 6F-LA and 6F-PEG as hydrophobic core and hydrophilic corona were synthesized through a pH sensitive linker, respectively. Their synthetic routes and chemical structures were shown in Additional file 1: Figures S1, S2 and confirmed by ^1^H-NMR and ^19^F-NMR, respectively.

**Fig. 2:**
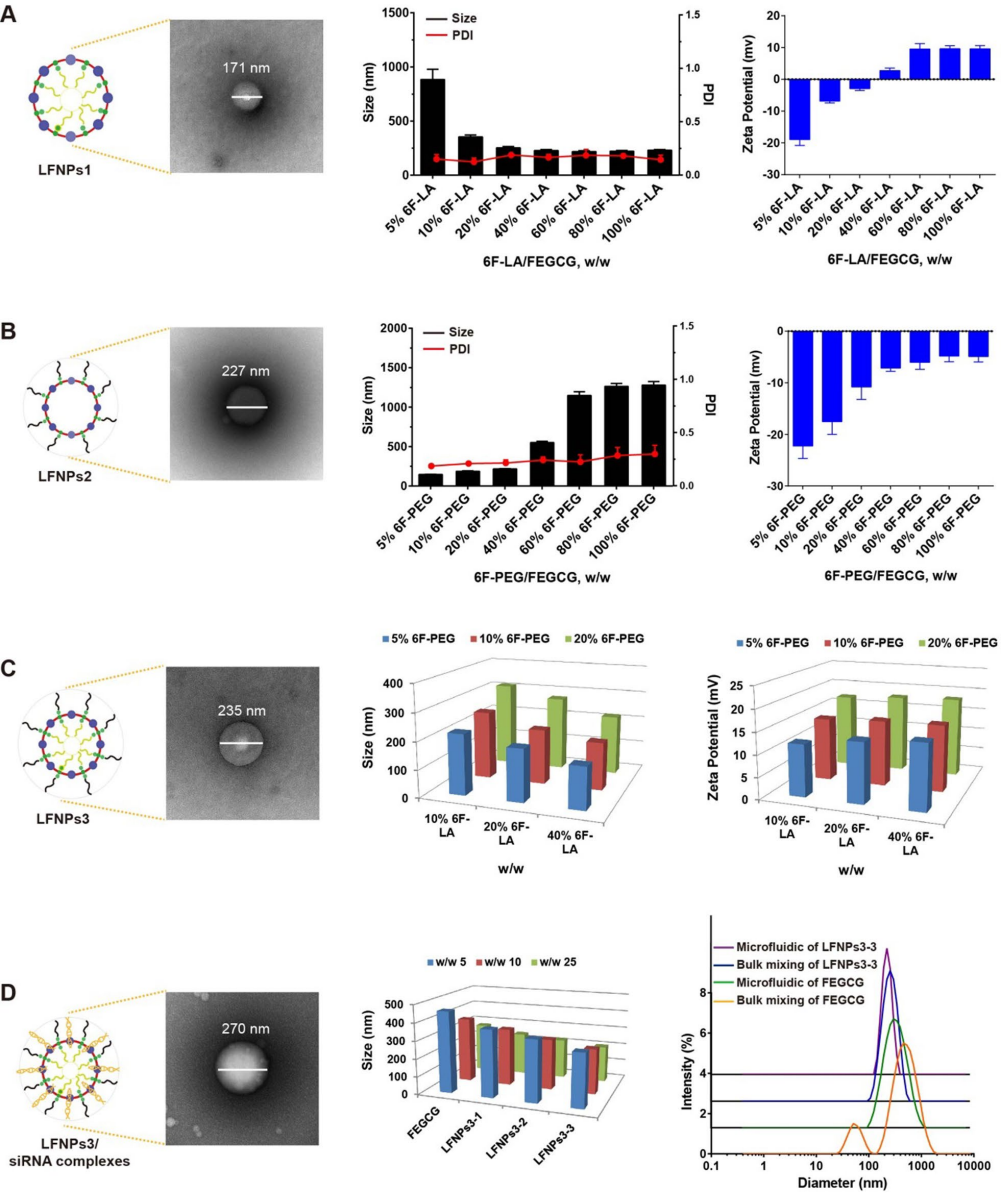
Preparation and characterization of LFNPs and LFNPs/siRNA complexes. The characterization of LFNPs1 **A**, LFNPs2 **B**, and LFNPs3 **C** determined by TEM, particle size (PDI), and zeta potential (n = 3). **D** The size distribution of different complexes prepared by microfluidic and manual mixing (n = 3)

The well-defined LFNPs within each sub-class were prepared by mixing FEGCG and 6F-LA/6F-PEG in the microfluidic platform. In detail, LFNPs1 with LA core were fabricated, and TEM image showed a spherical shape with the bright core. The particle size of LFNPs1 decreased gradually with increasing w/w ratio of 6F-LA/FEGCG from 883 nm at 5% 6F-LA to 229 nm at 100% 6F-LA. LFNPs2 with PEG corona were fabricated, and TEM image showed a spherical shape with the black corona. The particle size of LFNPs2 increased gradually with increasing w/w ratio of 6F-PEG/FEGCG from 144 nm at 5% 6F-PEG to 1278 nm at 100% 6F-PEG. The zeta potential of LFNPs1 and LFNPs2 was increased with increasing w/w ratio from − 18.9 and − 22 mV at w/w 5% to 9.6 and − 4.9 mV at w/w 100%, respectively. Both LFNPs1 and LFNPs2 displayed low PDI **(**Fig. 2A–B**)**.

LFNPs3 containing 6F-LA and 6F-PEG were next prepared, and the amount of 6F-LA and 6F-PEG was ranged from 10 to 40% and 5% to 20%, respectively. As shown in Fig. 2C, nine kinds of LFNPs3 were fabricated, and as anticipated, increased 6F-LA or decreased 6F-PEG content endowed LFNPs3 with smaller size, and their zeta potential was ranged from 12 to 18 mV. The representative TEM image confirmed the formation of LFNPs3. Furthermore, LFNPs3-1 with 10% 6F-LA and 20% 6F-PEG, LFNPs3-2 with 20% 6F-LA and 10% 6F-PEG, LFNPs3-3 with 40% 6F-LA and 5% 6F-PEG were screened for siRNA delivery study. The particle size of LFNPs3-3 complexes was smaller than FEGCG, LFNPs3-1, and LFNPs3-2 complexes, and increased w/w ratio further decreased their particle size. The zeta potential of produced complexes was negative ranged from − 1 mV to − 8 mV (Additional file 1: Figure S3). Extendedly, the method of manual mixing was adopted to compare the size distribution of FEGCG complexes and LFNPs3-3 complexes fabricated by microfluidic. The prepared complexes by microfluidic were almost monodispersed when compared with manual preparation, especially for FEGCG complexes **(**Fig. 2D**)**. These results demonstrate that the represented combinatorial approach accompanied with microfluidic device is an innovative technology for high-throughput screening of EGCG-based complexes, which will optimize the subsequent delivery of siTOX into solid tumors.

### Enhanced binding and trigged release of LFNPs/siRNA complexes in vitro

**Fig. 3:**
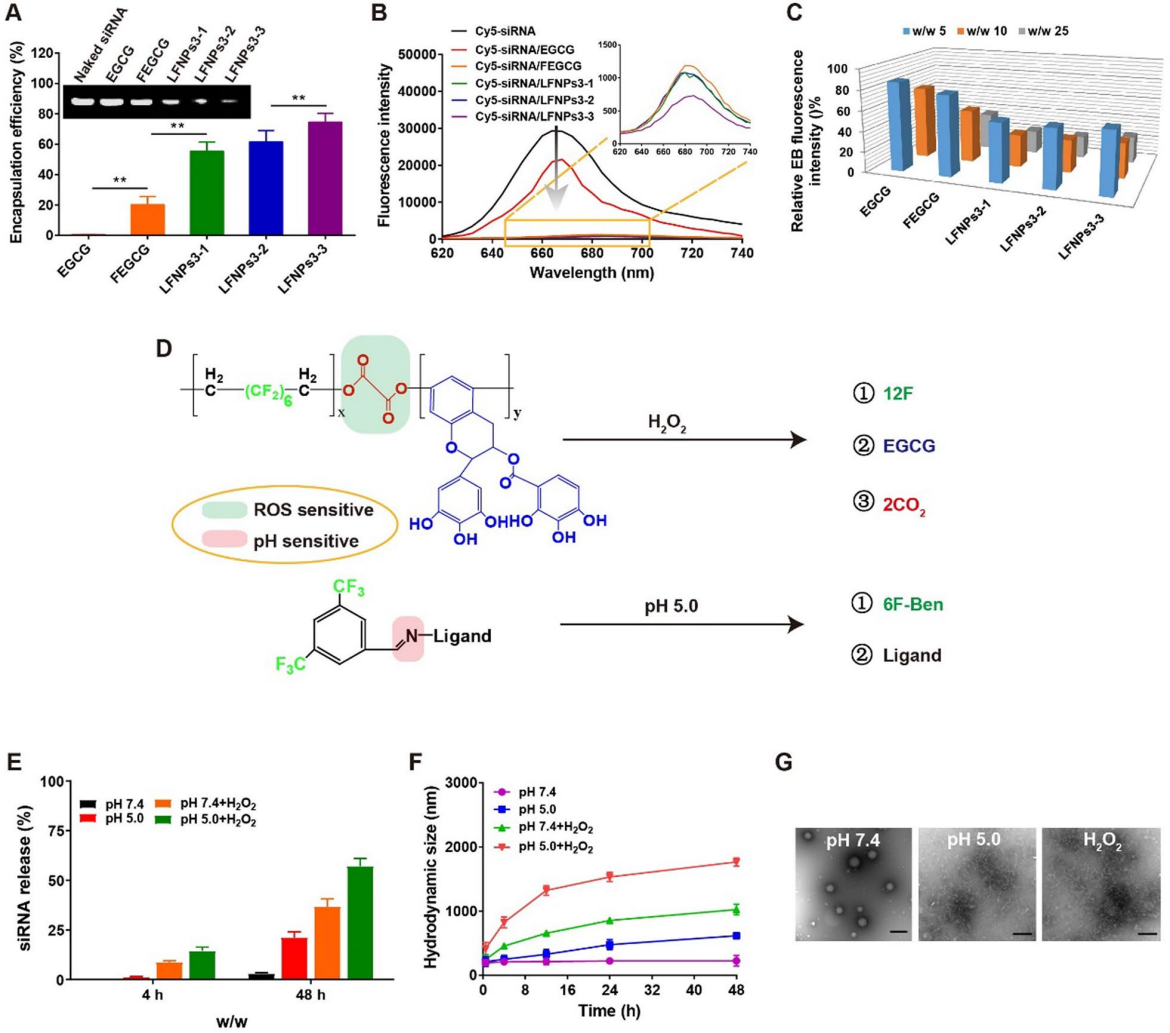
siRNA binding and sensitive release of LFNPs/siRNA complexes. **A**–**C** The assays of siRNA condense ability by EB competitive binding, Cy5-siRNA fluorescence quenching, and agarose gel electrophoresis (n = 3). (D) Schematic illustration of ROS/pH-triggered hydrolysis of LFNPs. (E–G) siRNA release profile, particle size, and morphology of LFNPs3-3 complexes incubated with different mediums (n = 3). **P < 0.01 by one-way analysis of variance (ANOVA) with Turkey’s multiple comparisons

The encapsulation efficiency of EGCG, FEGCG, and LFNPs3 to siRNA was first determined by agarose gel electrophoresis. As shown in Fig. 3A, LFNPs3-3 complexes displayed ~ fourfold higher encapsulation efficiency than FEGCG, and ~ 80-fold higher encapsulation efficiency than EGCG. Meanwhile, using Cy5-labeled siRNA as a tool, LFNPs3 complexes, especially LFNPs3-3 complexes, showed maximum fluorescence quenching in Cy5 **(**Fig. 3B**)**. In addition, the condense ability of LFNPs3 was further determined by ethidium bromide (EB) competitive binding assay. The interaction of EB and siRNA would yield a red fluorescence, and its changed signal could reflect the competitive binding of siRNA with carrier. At different w/w ratios, LFNPs3 showed stronger ability to decrease the signal value than FEGCG and EGCG **(**Fig. 3C**)**.

LFNPs complexes were expected to degrade rapidly in the high ROS as well as low pH environments to release the loaded siRNA **(**Fig. 3D**)**. To validate this dual response property, we treated LFNPs3-3 complexes with H_2_O_2_ incubation and/or adjusted the system to pH 5.0, and then monitored the change in Cy5 fluorescence intensity and particle size over time. Independent of H_2_O_2_ or pH 5.0 induced siRNA release to ~ 8.7% or ~ 1.4% at 4 h. Taken together, ~ 14.5% siRNA release could be observed when treated with both H_2_O_2_ and pH 5.0. Similarly, siRNA release rate increased to ~ 36.8% or ~ 21.2% at 48 h under H_2_O_2_ or pH incubation. Their combination further triggered siRNA release to ~ 57.1%. In addition, the increased particle size demonstrated that LFNPs3-3 complexes were unstable in H_2_O_2_ + pH conditions (Fig. 3E–F, Additional file 1: Figure S4A). In contrast, LFNPs3-3 complexes exhibited good stability against PBS and FBS conditions since no significant size change was observed (Additional file 1: Figure S4B). TEM images further confirmed ROS/pH trigged disassembly with changed morphology from spherical to irregular structures (Fig. 3G). These results suggest that the prepared LFNPs can deliver siTOX with satisfactory binding and sensitive release abilities.

### Biological functions of LFNPs/siRNA complexes in vitro

**Fig. 4:**
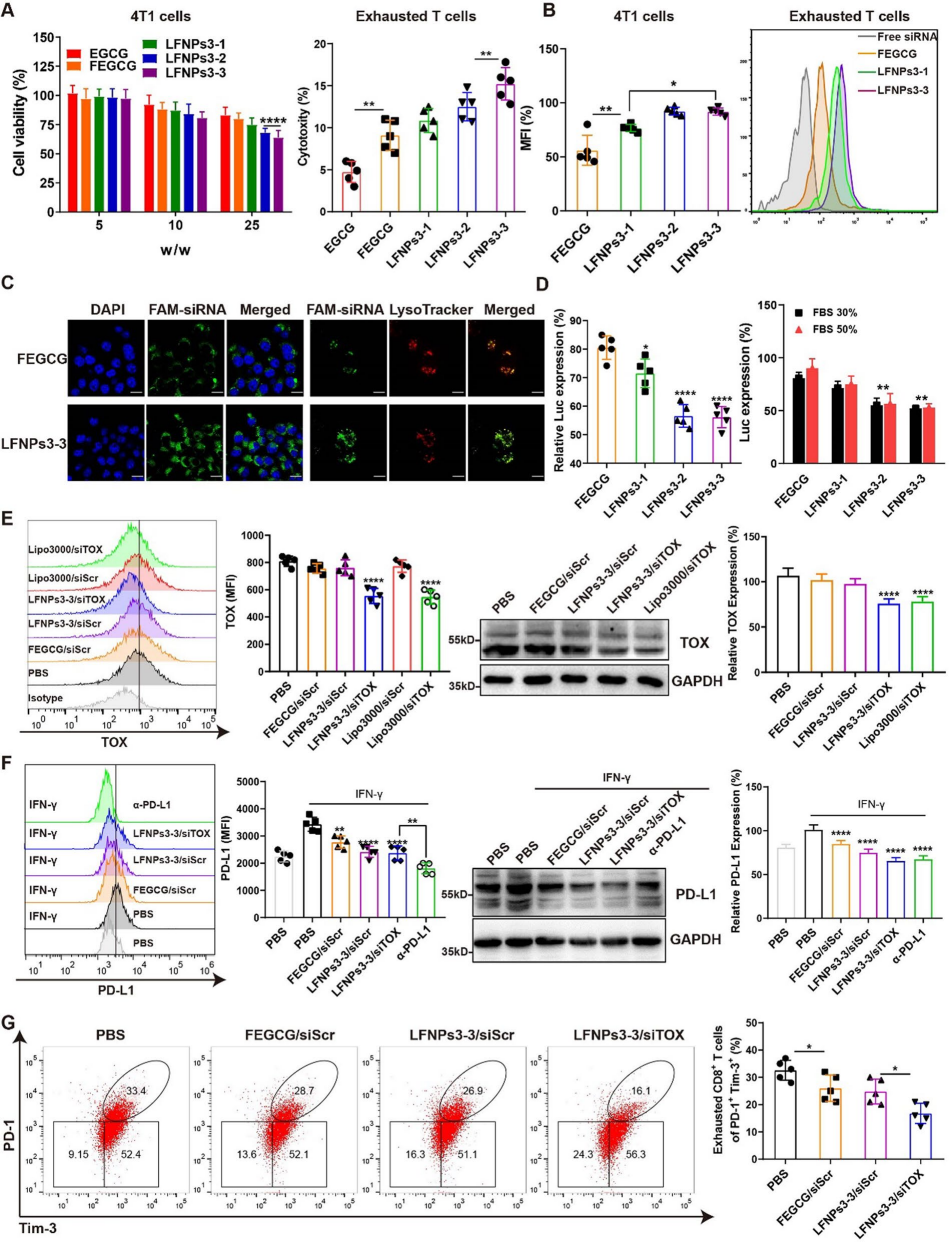
Regulatory functions of LFNPs/siRNA complexes in vitro. **A** Cytotoxicity of tested complexes in 4T1 cells and exhausted T cells (n = 5). **B** Cellular uptake of FEGCG complexes and different LFNPs3 complexes detected by flow cytometry in 4T1 cells and exhausted T cells (n = 5). **C** Cellular uptake and lysosomal escape of FEGCG complexes and LFNPs3-3 complexes detected by confocal microscopy. Scale bar = 10 µm. **D** Luc gene silencing of FEGCG complexes and different LFNPs3 complexes with or without FBS (n = 5). **E** TOX expression detected by flow cytometry and western blotting after different treatments in exhausted T cells (n = 5). **F** PD-L1 expression detected by flow cytometry and western blotting after different treatments in 4T1 cells (n = 5). **G** Percentage of PD-1 + Tim-3 + cells detected by flow cytometry after different treatments in exhausted T cells (n = 5). *P < 0.05, **P < 0.01, ****P < 0.0001 by one-way analysis of variance (ANOVA) with Turkey’s multiple comparisons

To evaluate the regulation of LFNPs/siTOX on PD-L1 expression of tumor cells and siTOX on the state of exhausted T cells, the cytotoxicity and siRNA delivery efficiency of LFNPs/siRNA complexes were first determined on 4T1 cells and exhausted T cells. As shown in Fig. 4A, LFNPs3 complexes showed the highest toxicity with the cell viability remaining about ~ 65% up to w/w 25. Meanwhile, their cytotoxicity was limited to less than 15% in T cells. Furthermore, LFNPs3-3 complexes achieved the highest cellular uptake than FEGCG complexes as evidenced on 4T1 and T cells. Improved cytosolic delivery and lysosomal escape of siRNA from LFNPs3-3 complexes were also visualized by confocal microscopy since the increased FAM fluorescence intensity and decreased colocalization between FAM and Lysotracker Red (Fig. 4B–C).

Choosing luciferase as a target, the transfection efficiency of siLuc was best achieved by using LFNPs3-2 and LFNPs3-3 as carriers in 4T1.Luc cells. The expected benefits on stability were evaluated by determining the transfection activity in different serum conditions. Unlike serum-free conditions, FEGCG complexes lost ~ 90% silencing activity at 50% FBS condition. In contrast, LFNPs formation boosted resistance of complexes to the serum (Fig. 4D). In addition, different LFNPs3-3 complexes named as manual and microfluidic complexes were prepared by manual mixing and microfluidic methods, respectively. Their stability, cellular uptake, and Luc silencing ability were compared, and the results showed that microfluidic complexes had higher stability, better cellular uptake, and lower gene expression than manual complexes (Additional file 1: Figure S5).

The expression of TOX in T cells and PD-L1 in 4T1 cells was examined, and the results showed that LFNPs3-3-based siTOX treatment successfully suppressed TOX expression, while the corresponding siScr complexes had no noticeable impact on TOX expression. Intriguingly, the efficacy of TOX silencing observed with LFNPs3-3 was comparable to that of the gold standard lipo3000, which was further verified through western blot analysis (Fig. 4E). In addition, anti-PD-L1 monoclonal antibody (α-PD-L1) as a positive control was used to validate the down-regulation of PD-L1 by FEGCG treatments. As shown in Fig. 4F, PD-L1 expression was increased after IFN-γ stimulation, while all test formulations led to a decrease in surface expression of PD-L1. As expected, α-PD-L1 exhibited the most significant silencing effect, although its silencing ability was similar to that of LFNPs3-3 in terms of total PD-L1 expression. Subsequently, the effect of LFNPs3-3/siTOX treatment on the state of exhausted T cells (PD1^+^ Tim3^+^) were examined, and the results demonstrated that all treatments especially LFNPs3-3/siTOX complexes decreased the percentage of PD-1^+^Tim-3^+^ cells from 33.4% in the PBS group to 16.1% (Fig. 4G). In addition, spleen-derived non-exhausted T cells were isolated to validate the gating strategy and subsequently induced into an exhausted phenotype using CD3/CD28 Dynabeads. As depicted in Additional file 1: Figure S6, the data demonstrated a noteworthy increase in the proportion of PD-1^+^ Tim-3^+^ cells, escalating from 6.4% to 67.0%. This augmentation can be considered as a characteristic feature of exhausted T cells. However, subsequent interventions alleviated the exhaustion state in T cells, as evidenced by the decreased percentage of PD-1^+^ Tim-3^+^ cells from 67.0% in the PBS group to 55.8% in the FEGCG/siScr group, 32.6% in the LFNPs3-3/siScr group, and 18.2% in the LFNPs3-3/siTOX group, respectively. The above results show that LFNPs3-3 can serve as a superior delivery system to synergistically regulate tumor cells and exhausted T cells in vitro.

### Biodistribution and therapeutic efficacy in vivo

**Fig. 5:**
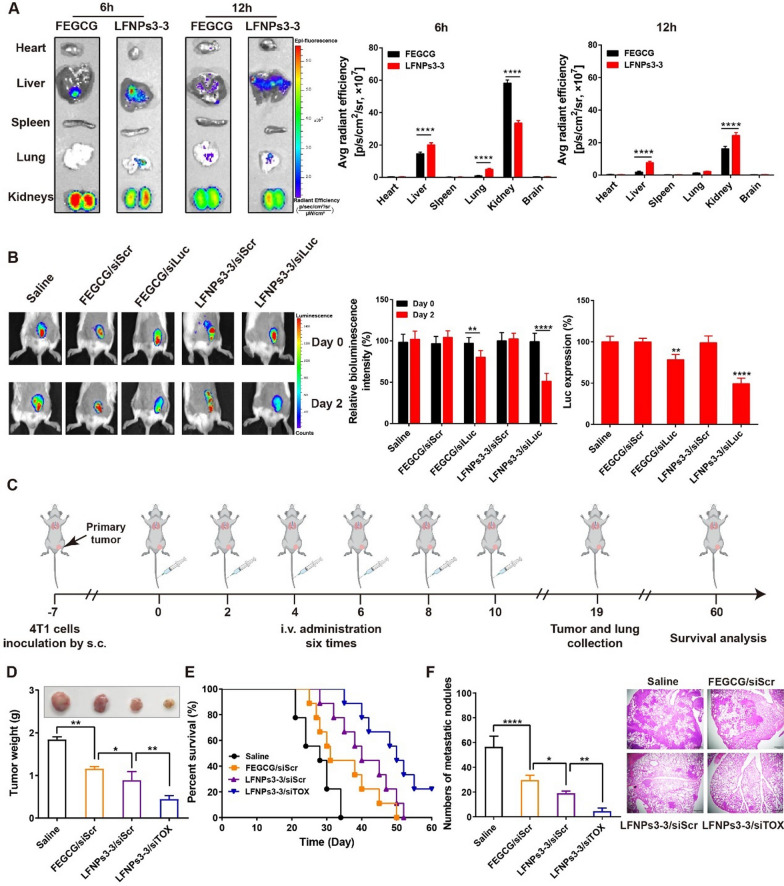
Biofunctions of LFNPs/siTOX complexes in vivo. **A** Representative organ distribution of Cy5-siRNA complexes at 6 h and 12 h postinjection, and the fluorescence intensity of Cy5-siRNA from ex vivo analysis (n = 3). **B** Luc gene silencing of FEGCG complexes and LFNPs3-3 complexes at day 0 and day 2, and the quantification of Luc expression, and ex vivo analysis of the Luc activity (n = 3). **C**–**D** Treatment schedule and tumor weight after different treatments with saline, FEGCG/siScr, LFNPs3-3/siScr, and LFNPs3-3/siTOX (n = 5). **E**–**F** Survival analysis and quantification of metastatic burden with reflected H and E staining (n = 9). *P < 0.05, **P < 0.01, ****P < 0.0001 by an unpaired two-tailed Student’s t-test or one-way analysis of variance (ANOVA) with Turkey’s multiple comparisons

A major challenge for siRNA complexes is that they are disassembled and cleared primarily through the glomerular basement membrane (GBM) of kidneys [33]. To compare the resistance of different LFNPs complexes to renal clearance, we assessed their stability in the presence of heparan sulfate (an anionic component of GBM) with FEGCG and EGCG complexes as controls. Using a fluorescence resonance energy transfer (FRET)-based assay, their ability to decrease susceptibility of heparin was in turn: LFNPs3-3 and 3–2 > LFNPs3-1 > FEGCG > EGCG complexes (Additional file 1: Figure S7). To further evaluate the stability of LFNPs complexes in vivo, the tissue biodistribution of Cy5-labeled LFNPs3-3 and FEGCG complexes were examined in healthy mice (Fig. 5A). Consistent with the result in vitro, LFNPs3-3 complexes exhibited lower accumulation in the kidneys relative to FEGCG complexes at 6 h postinjection. Additionally, the concentration of LFNPs3-3 complexes in liver and lung was higher than that of FEGCG complexes. At 12 h postinjection, LFNPs3-3 complexes exhibited higher accumulation in the liver and kidneys, and the total integrated fluorescence of all isolated organs was nearly twofold when compared with FEGCG complexes (Additional file 1: Figure S8).

Based on the improved stability of LFNPs3-3 complexes, the siRNA silencing ability of LFNPs3-3 complexes was evaluated through the model of 4T1.Luc expressed tumors (Fig. 5B). LFNPs3-3 or FEGCG complexes loaded with siLuc or siScr were injected into the mice by *i.v.*, and the bioluminescence images were captured at day 0 and day 2 postinjection. No Luc silencing was observed in siScr complexes treatment groups, validating that both polymers had no effects on Luc expression. On the contrast, diminished Luc expression was detected in FEGCG/siLuc complexes (~ 17%) and LFNPs3-3/siLuc complexes (~ 49%). This weaker signal in LFNPs3-3/siLuc complexes was further confirmed by determining Luc activity in excised tumor tissues. The above results demonstrate that LFNPs3-3 loaded siRNA complexes possess good stability, leading to satisfactory target silencing ability in tumors.

The anti-tumor activity of LFNPs3-3/siTOX complexes was then evaluated, and the treat regimen was shown in Fig. 5C. Different complexes of FEGCG/siScr, LFNPs3-3/siScr, and LFNPs3-3/siTOX with saline group as a control were administered on days 0, 2, 4, 6, 8, and 10 for total of six times. Some mice were sacrificed on day 19, while the remaining mice continued to be used for survival analysis. As shown in Fig. 5D–E (Additional file 1: Figure S9), FEGCG/siScr or LFNPs3-3/siScr treatment had generated significant tumor inhibition, and the augmented therapeutic effect could be achieved in LFNPs3-3/siTOX treatment. The median survival time was prolonged from 28 day in saline group to 31 day of FEGCG/siScr group, 40 day of LFNPs3-3/siScr group, and 50 day of LFNPs3-3/siTOX group. Taken together, the burden of lung metastasis was also significantly ameliorated after main treatments, and the related antimetastatic efficacy was further demonstrated in H&E staining (Fig. 5F). The body weight didn’t show obvious change in different treatments (Additional file 1: Figure S10). Furthermore, the comparison of anti-tumor efficacy between FEGCG/siTOX and LFNPs3-3/siTOX further underscored the superiority of the LFNPs3-3/siTOX combination (Additional file 1: Figure S11). These results provide compelling evidence for the stability and targeted delivery capabilities of LFNPs3-3 as a delivery system in tumor. Moreover, they support the significant anti-tumor and antimetastatic effects achieved through the synergistic action of LFNPs3-3 and siTOX.

### Immune activation and biosafety in vivo

**Fig. 6:**
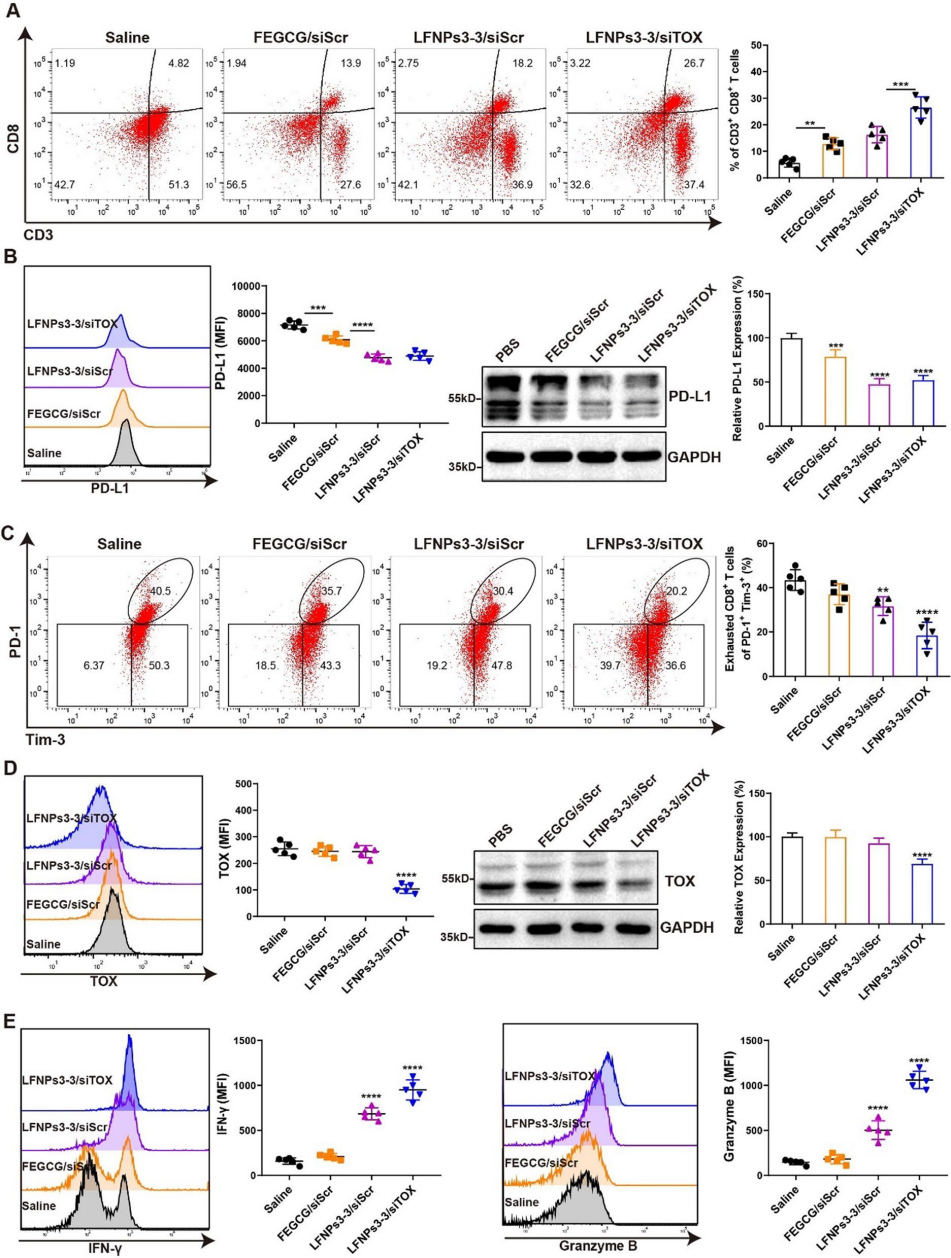
Immune activation mechanism in vivo. **A** Percentage and statistical analysis of CD3 + CD8 + T cells detected by flow cytometry (n = 5). **B** PD-L1 expression detected by flow cytometry and western blotting after different treatments (n = 5). **C** Percentage and statistical analysis of Tim3 + PD-1 + cells detected by flow cytometry (n = 5). **D** TOX expression detected by flow cytometry and western blotting after different treatments (n = 5). **E** Cytokines of IFN-γ and granzyme B in CD8 + T cells detected by flow cytometry (n = 5). **P < 0.01, ***P < 0.001, ****P < 0.0001 by one-way analysis of variance (ANOVA) with Turkey’s multiple comparisons

To reveal the mechanism of immune activation, T cell infiltration as a crucial index was first investigated by determining the changed ratio of CD8^+^ T (CD3^+^ CD8^+^) cells. As shown in Fig. 6A, the ratio of CD8^+^ T cells increased to 26.7% of LFNPs3-3/siTOX treatment, higher than 4.8% of saline group, 13.9% of FEGCG/siScr treatment, and 18.2% of LFNPs3-3/siScr treatment. PD-L1 expression and the state of exhausted CD8^+^ T cells were next evaluated. Figure 6B provided evidence of PD-L1 knockdown in vivo through flow cytometry and western blot analysis. All treatments, especially LFNPs3-3/siScr and LFNPs3-3/siTOX treatments, showed positive effects in reducing PD-L1 expression. By counting the proportion of Tim-3^+^PD-1^+^ cells, LFNPs3-3/siTOX treatment significantly reduced the percentage of Tim-3^+^PD-1^+^ cells from 40.5% of saline group to 20.2%. Meanwhile, LFNPs3-3/siScr treatment could also effectively reduce the percentage of Tim-3^+^PD-1^+^ cells to 30.4% (Fig. 6C). Several studies had revealed that TOX was up-regulated in breast cancer, and its high level was associated with the poor prognosis [34, 35]. As shown in Fig. 6D, only siTOX treatment exhibited a significant reduction in TOX expression, as evidenced by both flow cytometry and western blot data. In addition, LFNPs3-3 treatments, specifically LFNPs3-3/siTOX, have been observed to enhance the release of IFN-γ and granzyme B in CD8^+^ T cells. Finally, the tumor necrosis factor-α (TNF-α) and IFN-γ cytokines were tested, and their elevated level was reflected in main treatments (Additional file 1: Figure S12). The increased level of these cytotoxic cytokines signified the restoration of T cell functionality, thus indicating an improvement in overall immune response.

EGCG is a powerful antioxidant with great therapeutics in different diseases because of its safety and bioavailability [36]. Therefore, we evaluated the biosafety of FEGCG and LFNPs3-3 complexes on healthy mice after *i.v.* injection. As shown in Additional file 1: Figure S13, main organs including heart, liver, spleen, lung and kidneys were collected, and H&E staining demonstrated that both treatments didn’t induce a significant change in organ morphology when compared to the saline group. Based on the major accumulation of siRNA complexes in liver and kidneys, alanine aminotransferase (ALT) and aspartate aminotransferase (AST) as liver injury indicators, blood urea nitrogen (BUN) and creatinine (CR) as kidney injury indicators, were selected to observe their effect on liver and kidney functions. As depicted in Additional file 1: Figure S14, both administrations did not cause the change of ALT, AST, BUN and CR levels compared with the saline group, proving the biosafety of FEGCG and the constructed LFNPs. In addition, the effect of different complexes on hemolysis were further evaluated, and the results showed that all formulation did not induce significant hemolysis compared with the positive control of Triton with 100% hemolysis rate (Additional file 1: Figure S15). These results indicated that the combination of LFNPs and siTOX could effectively inhibit PD-L1 expression and attenuate CD8^+^ T cells exhaustion to increase CD8^+^ T infiltration, finally leading to the robust immune activation while possessing good safety and hemocompatibility.

## Discussion

Green tea is a widely consumed beverage that has firmly integrated into our daily lives. However, the clinical application of EGCG derived from green tea is limited due to its low bioavailability and poor chemical stability. To overcome these limitations, structural modification and nanotechnology have been employed as effective strategies [22, 37]. By combining fluorine modification and nano-formulation, we developed LFNPs composed of FEGCG, 6F-LA, and 6F-PEG for the purpose of delivering siRNA. Our results have demonstrated the superiority of LFNPs in terms of enhanced siRNA binding, as well as their ROS/pH sensitivity and size stability (Fig. 3). In addition, they also exhibited good safety and hemocompatibility, taking into consideration the potential toxicity and immunogenicity of nano-carriers (Additional file 1: Figure S13–15). However, it is worth noting that further research is still required to fully explore the therapeutic potentials of LFNPs, particularly in relation to their impact on the bioavailability and stability of EGCG.

Microfluidics is a versatile technology that allows for precise control over the size, size dispersity, and surface chemistry of nanoparticles, making it widely used in their synthesis to ensure consistent quality and stability. In comparison to the conventional manual mixing method, complexes of LFNPs3-3 prepared using microfluidics exhibited enhanced stability, improved cellular uptake, and reduced Luc expression (Additional file 1: Figure S5). These findings clearly demonstrate the significant advantages of microfluidics over manual operations when it comes to synthesizing nanoplatforms with uniform physicochemical properties and sufficient quantities. This can be attributed to the ability of microfluidics to meticulously regulate the processes of nano-nucleation, growth, and aggregation. Moving forward, future research will explore alternative operations and devices to maximize the efficacy of LFNPs complexes.

The efficient delivery of siRNA in vivo relies on its prolonged circulation time in the bloodstream [38, 39]. To enhance this circulation time, siRNA polyplexes are often surface-modified with PEG. Among different LFNPs, LFNPs3-3 complexes, which contain fluorine, PEG, and hydrophobic modifications, exhibit superior resistance to kidney filtration, higher organ accumulation, and enhanced Luc silencing properties compared to FEGCG complexes with fluorine modification alone (Fig. 5A–B). However, the use of PEG-containing formulations can lead to the production of anti-PEG IgM after the first injection, resulting in a reduced blood circulation of subsequent doses, known as the accelerated blood clearance (ABC) phenomenon. Previous studies have shown that the production of anti-PEG IgM is significantly lower for PEG-coated siRNA-lipoplex compared to PEG-coated naked cationic liposomes. Furthermore, the anti-PEG IgM response is inversely correlated with the dose of PEG-coated siRNA-lipoplex [40–42]. Although the safety of LFNPs and siTOX-loaded complexes may reduce the anti-PEG IgM response, further investigation is required to determine their specific mechanisms.

Many creative strategies have been practiced to evoke the immune response in immunotherapy, while the low response rate remains the most worrying among different tumors [8, 23]. Therefore, we propose a novel strategy to resolve the aforementioned dilemma by simultaneously regulating tumor cells and exhausted T cells based on potential targets of PD-L1 and siTOX. The prepared LFNPs3-3/siTOX complexes can effectively inhibit the expression of PD-L1 and TOX to mitigate T exhaustion, finally leading to the superior antitumor and antimetastatic efficacy by eliciting a robust immune activation (Fig. 6). In addition, we found that oxidized EGCG is more likely to form NPs with monoclonal antibody in our other study. Therefore, our novel EGCG based delivery system with PD-(L)1 monoclonal antibody will be designed to validate its anti-tumor efficacy in future work.

In general, the various constituents present in LFNPs complexes, namely EGCG, fluorine, PEG, LA, and siTOX, boast high safety and biocompatibility. From a scientific standpoint, the large-scale production of LFNPs complexes can be enabled by microfluidics and the affordability and ease of access to each individual component. Following the completion of large-scale production, the safety and effectiveness of LFNPs/siTOX complexes can be assessed on a larger scale, utilizing animal models such as monkeys or pigs. If successful, this would signify its readiness for clinical application. Ultimately, we developed a functional EGCG-based siRNA delivery system, presenting a promising strategy for transforming ‘‘cold’’ tumors into ‘‘hot’’ tumors in cancer immunotherapy.

### Supplementary Information

**Additional file 1: Figure S1.** **A** The synthesis route of 6F-LA and 6F-PEG. **B** ^1^H-NMR spectrum of 6F-LA and 6F-PEG. **Figure S2.** ^19^F-NMR spectrum of 6F-LA and 6F-PEG. **Figure S3.** The zeta potential of tested complexes (n=3). **Figure S4.** **A** Cy5 fluorescence spectra of LFNPs3-3/Cy5-siRNA complexes under different conditions at 4 h and 48 h. (B) Stability of LFNPs3-3 complexes against PBS (pH 7.4), 10% FBS or their combination (n=3). **Figure S5.** Stability, cellular uptake, and Luc expression of manual complexes and microfluidic complexes (n=5). *P < 0.05, ***P < 0.001 by an unpaired two-tailed Student’s t-test. **Figure S6.** Percentage of PD-1^+^Tim-3^+^ cells detected by flow cytometry after different treatments in spleen-derived non-exhausted T cells with/without CD3/CD28 stimulation (n=5). *P < 0.01, ****P < 0.0001 by one-way analysis of variance (ANOVA) with Turkey’s multiple comparisons. **Figure S7.** Stability of different complexes against disassembly with heparin (n=3). **Figure S8.** Total Cy5-siRNA retention at 12 h postinjection determined from isolated organs (n=3). **P < 0.01 by an unpaired two-tailed Student’s t-test. **Figure S9.** The tumor growth curves of individual mouse in different treatments (n=5). **Figure S10.** Lung image and body weight change (n=5). **Figure S11.** Tumor image, tumor volume, and tumor weight after different treatments with saline, FEGCG/siTOX, and LFNPs3-3/siTOX (n=5). *P < 0.05, **P < 0.01 by one-way analysis of variance (ANOVA) with Turkey’s multiple comparisons. **Figure S12.** TNF-α and IFN-γ levels in plasma after different treatments (n=5). *P < 0.05, ****P<0.01 by one-way analysis of variance (ANOVA) with Turkey’s multiple comparisons. **Figure S13.** H&E staining of major organ tissues in healthy mice. **Figure S14.** Serum biomarkers levels of ALT, AST, BUN and CR (n=5). **Figure S15.** Hemolysis rate of FEGCG/siScr, LFNPs3-3/siScr, and LFNPs3-3/siTOX complexes with Triton as positive control in red blood cells (n=5).

## Data Availability

The data used in this study are included in this article and are available from the corresponding authors on reasonable request.
