# Supplementary material for: Microfluidics-enabled fluorinated assembly of EGCG-ligands-siTOX nanoparticles for synergetic tumor cells and exhausted t cells regulation in cancer immunotherapy
Source: J Nanobiotechnology. 2024 Mar 4;22:90. doi: 10.1186/s12951-024-02328-4 (PMC10910710; doi:10.1186/s12951-024-02328-4)
Supplement: Supplementary file 1 — Additional file 1: Figure S1. A The synthesis route of 6F-LA and 6F-PEG. B 1H-NMR spectrum of 6F-LA and 6F-PEG. Figure S2. 19F-NMR spectrum of 6F-LA and 6F-PEG. Figure S3. The zeta potential of tested complexes (n=3). Figure S4. A Cy5 fluorescence spectra of LFNPs3-3/Cy5-siRNA complexes under different conditions at 4 h and 48 h. (B) Stability of LFNPs3-3 complexes against PBS (pH 7.4), 10% FBS or their combination (n=3). Figure S5. Stability, cellular uptake, and Luc expression of manual complexes and microfluidic complexes (n=5). *P < 0.05, ***P < 0.001 by an unpaired two-tailed Student’s t-test. Figure S6. Percentage of PD-1+Tim-3+ cells detected by flow cytometry after different treatments in spleen-derived non-exhausted T cells with/without CD3/CD28 stimulation (n=5). *P < 0.01, ****P < 0.0001 by one-way analysis of variance (ANOVA) with Turkey’s multiple comparisons. Figure S7. Stability of different complexes against disassembly with heparin (n=3). Figure S8. Total Cy5-siRNA retention at 12 h postinjection determined from isolated organs (n=3). **P < 0.01 by an unpaired two-tailed Student’s t-test. Figure S9. The tumor growth curves of individual mouse in different treatments (n=5). Figure S10. Lung image and body weight change (n=5). Figure S11. Tumor image, tumor volume, and tumor weight after different treatments with saline, FEGCG/siTOX, and LFNPs3-3/siTOX (n=5). *P < 0.05, **P < 0.01 by one-way analysis of variance (ANOVA) with Turkey’s multiple comparisons. Figure S12. TNF-α and IFN-γ levels in plasma after different treatments (n=5). *P < 0.05, ****P<0.01 by one-way analysis of variance (ANOVA) with Turkey’s multiple comparisons. Figure S13. H&E staining of major organ tissues in healthy mice. Figure S14. Serum biomarkers levels of ALT, AST, BUN and CR (n=5). Figure S15. Hemolysis rate of FEGCG/siScr, LFNPs3-3/siScr, and LFNPs3-3/siTOX complexes with Triton as positive control in red blood cells (n=5). [file 12951_2024_2328_MOESM1_ESM.docx]

**Additional file Information**

**Microfluidics-enabled Fluorinated Assembly of EGCG-ligands-siTOX Nanoparticles for Synergetic Tumor cells and Exhausted T cells Regulation in Cancer Immunotherapy**

Xiaowei Han^1,2†^, Guozheng Zhang^1†^, Xiaozhen Wu^3^, Shufeng Xu^1^, Jiahuan Liu^1^, Kaikai Wang^3^, Tianqing Liu^4^*, Pengkai Wu^2^*

^1^Department of Radiology, The Quzhou Affiliated Hospital of Wenzhou Medical University, Quzhou People's Hospital, Quzhou, China.

^2^Department of Hepatobiliary Surgery, Innovative Institute of Tumor Immunity and Medicine (ITIM), Anhui Province Key Laboratory of Tumor Immune Microenvironment and Immunotherapy, The First Affiliated Hospital of Anhui Medical University, Hefei, 230022, China.

^3^School of Pharmacy, Nantong University, Nantong 226001, China

^4^NICM Health Research Institute, Western Sydney University, Sydney, NSW 2145, Australia

*Corresponding author (Pengkai Wu, email: wupengkai@ahmu.edu.cn; Tianqing Liu, email: [M.Liu3@westernsydney.edu.au](mailto:M.Liu3@westernsydney.edu.au))

^†^Xiaowei Han and Guozheng Zhang contributed equally to this work

**Materials and Methods**

**Materials**

The following reagents were purchased for this study: oxalyl chloride solution, 3,5-Bis(trifluoromethyl)benzaldehyde (6F-Ben), and 12-Aminolauric Acid (LA) from Aladdin Bio-Chem Technology Co., Ltd (Shanghai, China); (−)-epigallocatechin-3-O-gallate (EGCG) from Energy Chemical (Shanghai, China); polyethylene glycol (PEG5000-NH_2_) from Pengsheng Biotechnology Co., Ltd (Shanghai, China); 2,2,3,3,4,4,5,5,6,6,7,7-Dodecafluoro-1,8-octanediol (12F) and 2-Mercaptoethano (M6230) from Macklin Biochemical Co., Ltd (Shanghai, China); 2-oxopropanoic acid sodium salt (S8636) from Sigma-Aldrich Co., Ltd (USA); the recombinant mouse IL-2 (Cat# 575404), Ultra-LEAF™ Purified anti-mouse PD-L1 Antibody (Cat# 124318), anti-CD45-APC (Cat# 103111), anti-CD8-BV510 (Cat#100751), anti-Tim-3-PE (Cat#119704), anti-Tim3-PE-Cy7 (Cat#119716), anti-Ki67-FITC (Cat#652410), anti-Granzyme B-Alexa Fluor@647 (Cat#515405), and anti-IFN-γ-FITC (Cat#505806) from BioLegend; the Dynabeads™ Mouse T-Activator CD3/CD28 (Cat#11452D), Lipofectamine® 3000 (lipo3000, Cat#L3000001), anti-CD3-PE (Cat#12003182), anti-PD-1-APC-Cy7 (Cat#47998582), anti-IgG2a Isotype-eFluor™ 660 (Cat#50432182), and anti-TOX-eFluor™ 660 (Cat#50650282) from Thermo Fisher Scientific; the anti-PD-1-PE-Cy7 (Cat#25-9985-82) from BD Pharmingen; the anti-PD-L1-PE (Cat#E-AB-F1132D) from Elabscience (Wuhan, China). TNF-α (Cat#EK0527) and IFN-γ (Cat#EK0375) ELISA kits from Boster Biological Technology Co. Ltd. (Wuhan, China). FAM, Cy3, and Cy5 labeled siRNA, and siScr (sense strand, 5’-UUC UCC GAA CGU GUC ACG UTT-3’), siLuc (sense strand, 5’-GGA CGA GGA CGA GCA CUU CUU-3’), siTOX (sense strand, 5’-GGA CAA AGC ACU GUA CCU UTT-3’) from GenePharma Co., Ltd (Shanghai, China). Beyotime Biotechnology Co., Ltd (Shanghai, China) provided all other reagents, unless stated otherwise.

**Cell lines and culture**

The cell lines of 4T1 (murine breast cancer cells) and Luciferase-expressed 4T1 (4T1-Luc) were purchased from the American Type Culture Collection and cultured in RPMI 1640 with 10% FBS (Gibco, USA) and 1% penicillin-streptomycin solution (Wisent, China). To obtain exhausted CD8^+^ T cells, the 4T1 tumor was excised to digest using collagenase II, and then lymphocytes were separated from tumor tissues using a gradient Percoll method with concentrations of 80% and 40% (Biosharp, China). Exhausted CD8^+^ T cells were further purified from the above lymphocytes using the CD8 Positive Selection Kit (STEMCELL, Canada). To obtain non-exhausted T cells, the lymphocytes from spleen were isolated using a Lymphocyte Separation Medium (Dakewe, China). T cells were further purified from the above lymphocytes using the MojoSort™ Mouse CD3 T Cell Isolation Kit (BioLegend, USA). These T cells were cultured in RPMI 1640 medium supplemented with 10% FBS, 1% penicillin-streptomycin, 2-Mercaptoethano (55 µM), HEPES (10 mM), 2-oxopropanoic acid sodium salt (1 mM), and IL2 (20 ng/mL). In some case, these non-exhausted T cells were activated and stimulated to an exhausted phenotype by adding CD3/CD28 Dynabeads twice for 3 days each time (25 µL for 10^6^ cells). All cells were maintained in a 37°C incubator with 5% CO_2_.

**Animals and tumor models**

The female Balb/c mice (5 weeks old) were obtained from Gempharmatech Co., Ltd. The tumor growth and metastatic spread of 4T1 cells in Balb/c mice closely resemble that of human breast cancer. Upon injection into Balb/c mice, 4T1 cells exhibit a remarkable propensity for inducing highly metastatic tumors, which can disseminate to various organs including the lung, liver, lymph nodes, and brain. Therefore, the tumor model was constructed by subcutaneous injection of 4T1(Luc) cells (1×10^6^ per mouse) into the mice mammary fat pad on day -7 with or without intravenous injection of 4T1 cells (2×10^5^ per mouse) on day 6. All experimental procedures involving animals were conducted in accordance with the guidelines provided by the Animal Care and Use Committee of the First Affiliated Hospital of Anhui Medical University.


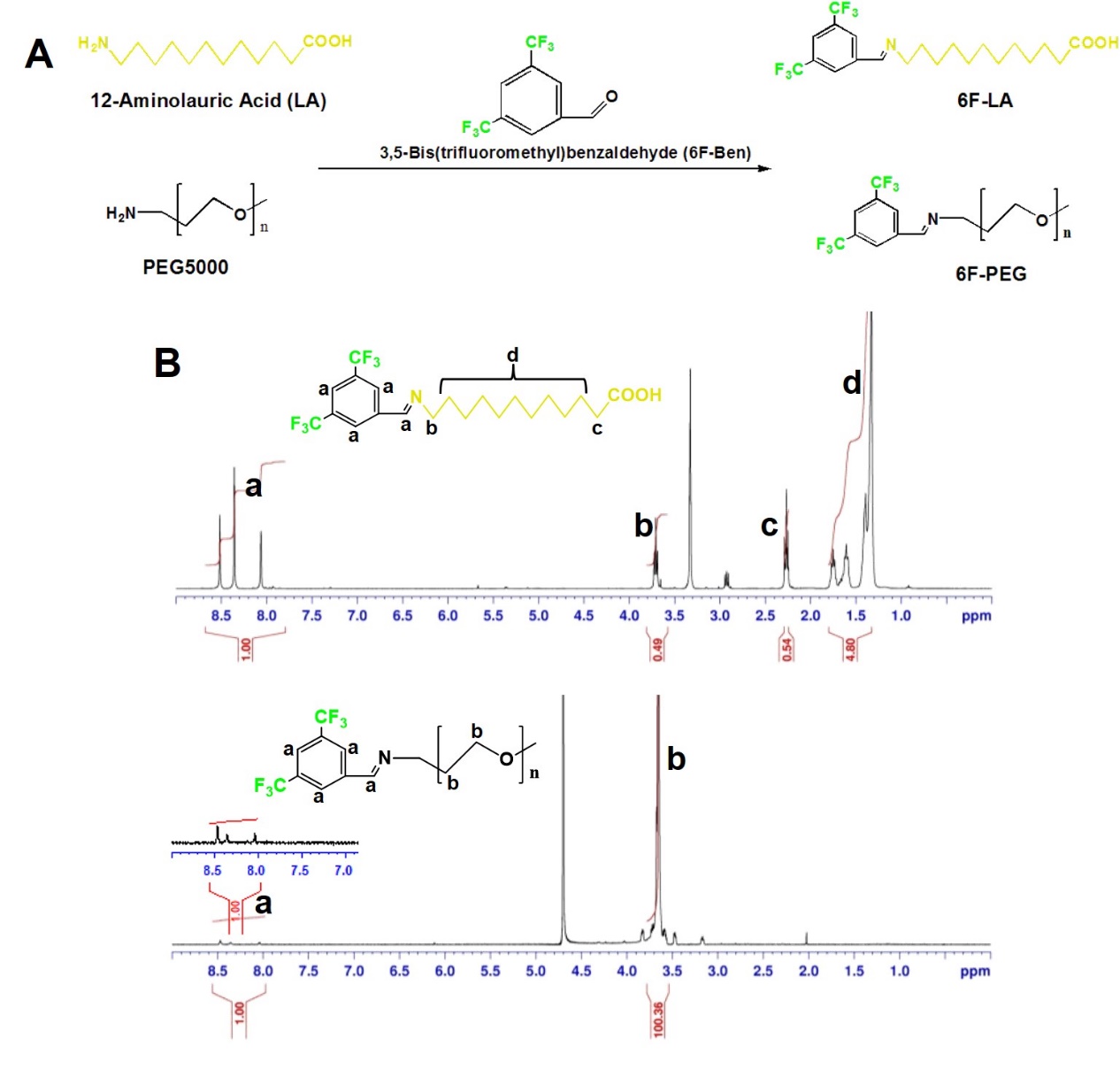


**Figure S1**. (A) The synthesis route of 6F-LA and 6F-PEG. (B) ^1^H-NMR spectrum of 6F-LA and 6F-PEG.


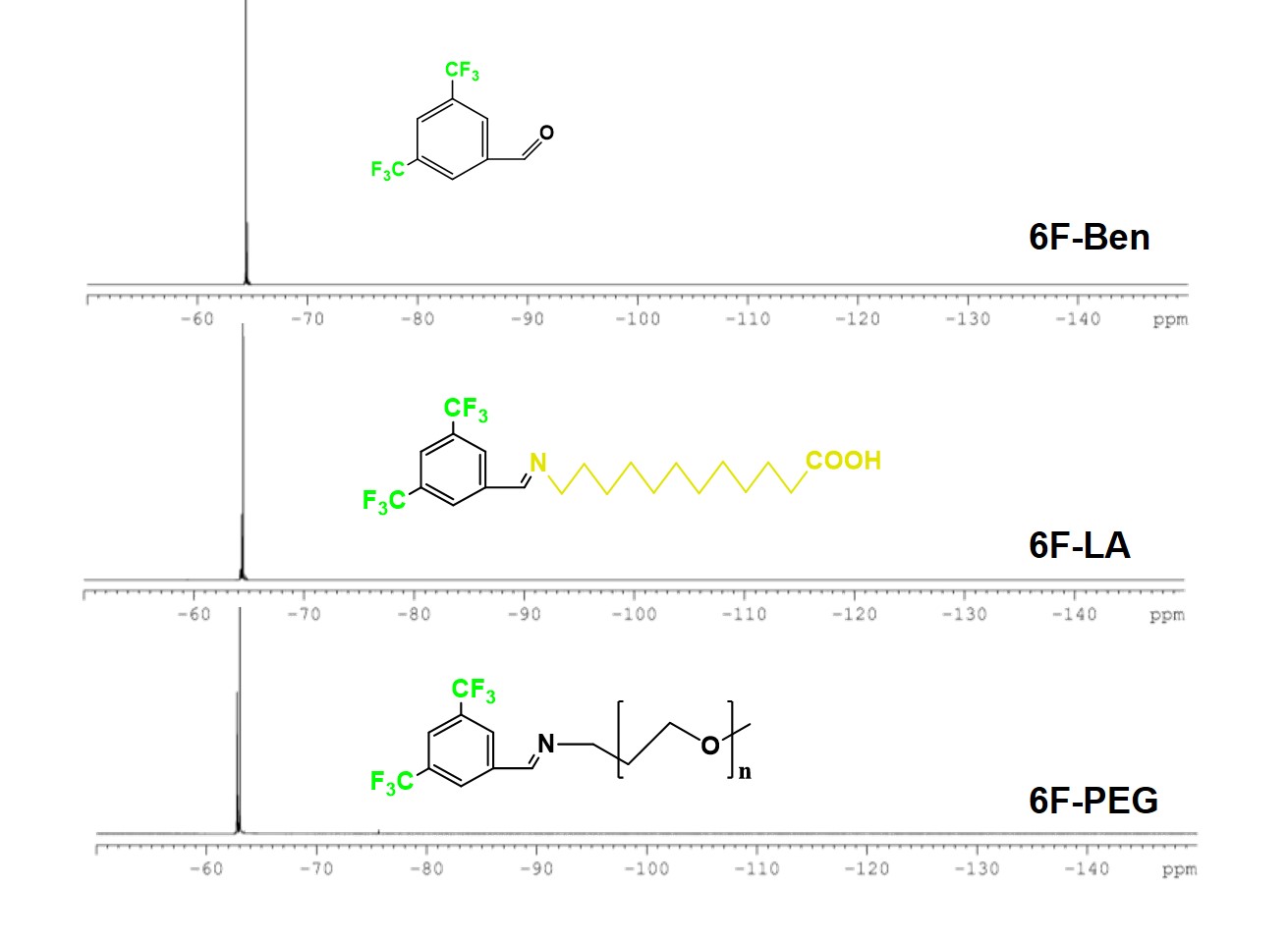


**Figure S2**. ^19^F-NMR spectrum of 6F-LA and 6F-PEG.


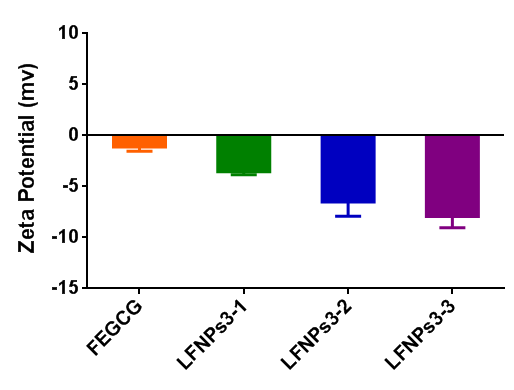


**Figure S3**. The zeta potential of tested complexes (n=3).


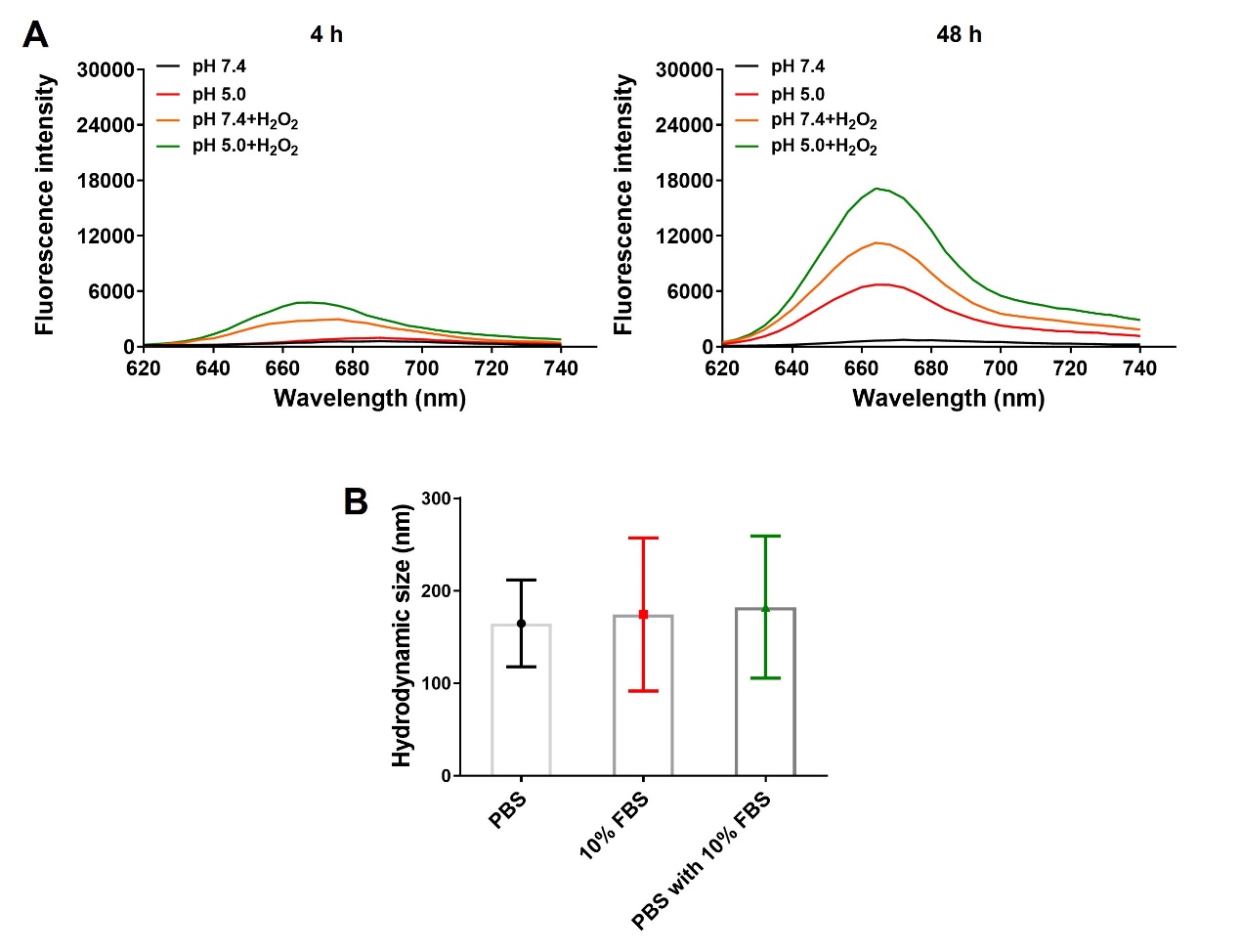


**Figure S4**. (A) Cy5 fluorescence spectra of LFNPs3-3/Cy5-siRNA complexes under different conditions at 4 h and 48 h. (B) Stability of LFNPs3-3 complexes against PBS (pH 7.4), 10% FBS or their combination (n=3).


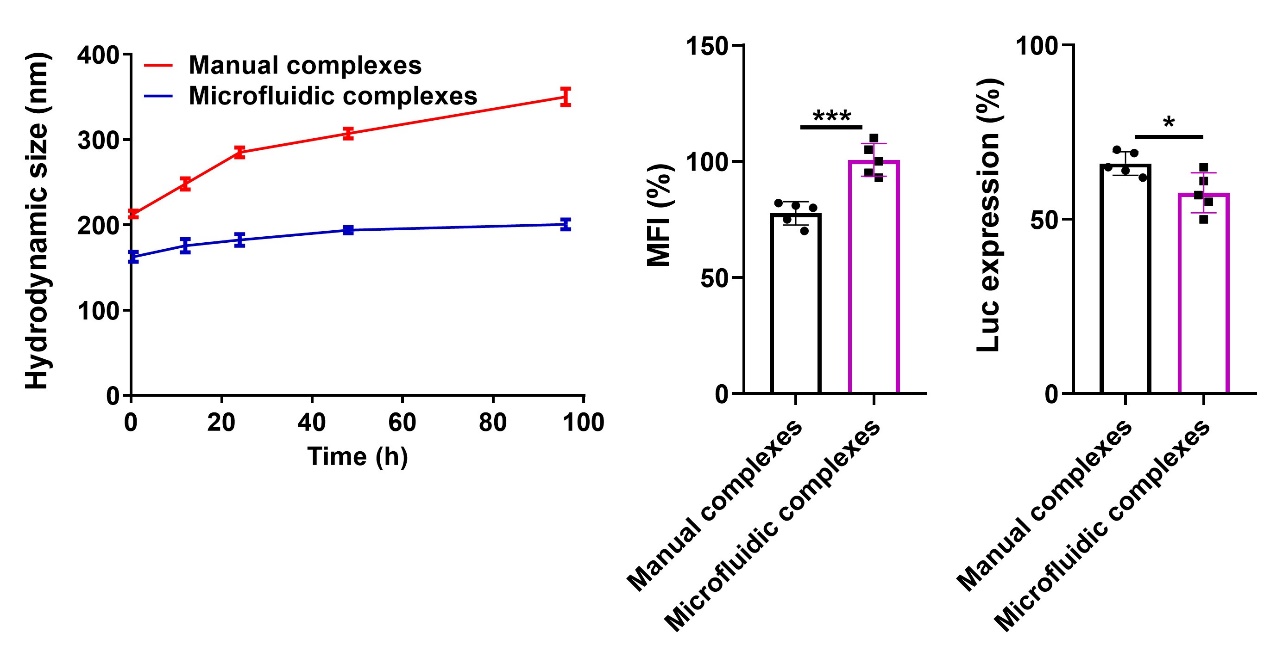


**Figure S5**. Stability, cellular uptake, and Luc expression of manual complexes and microfluidic complexes (n=5). *P < 0.05, ***P < 0.001 by an unpaired two-tailed Student’s t-test.


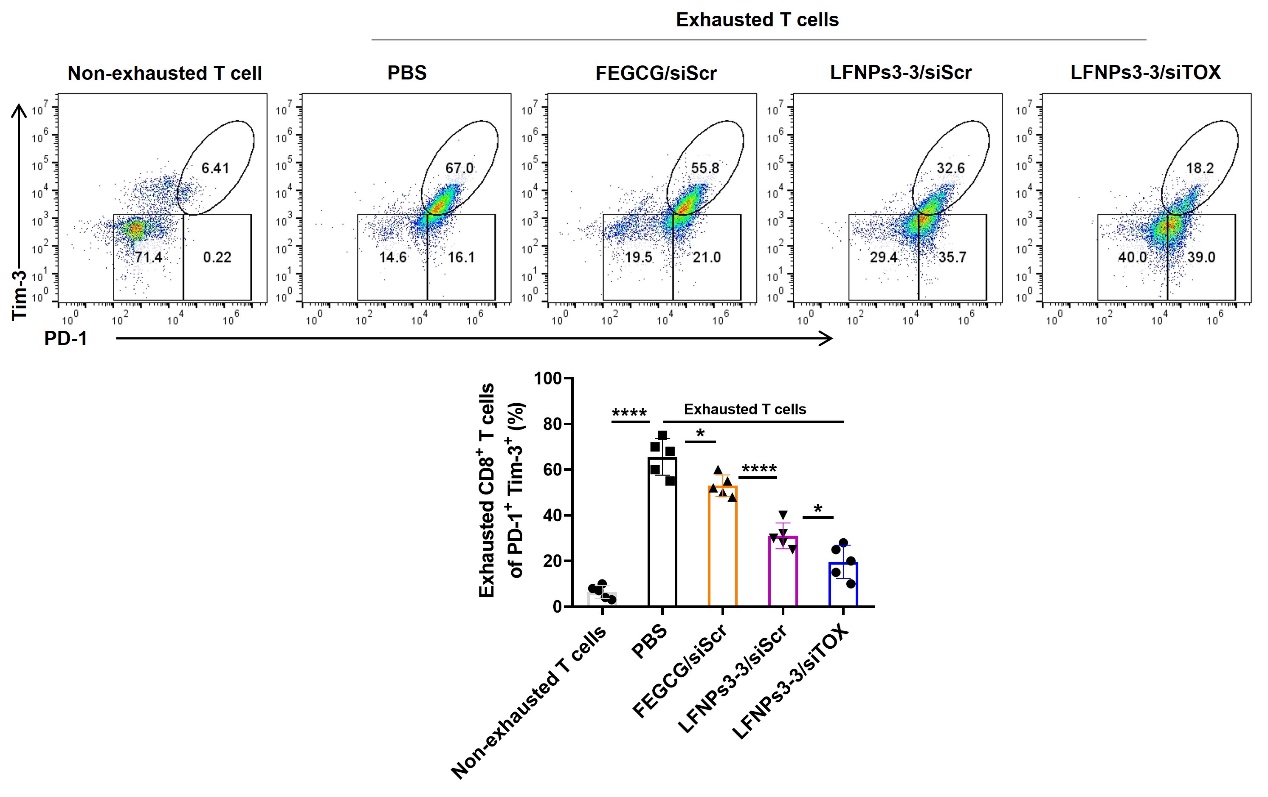


**Figure S6**. Percentage of PD-1^+^Tim-3^+^ cells detected by flow cytometry after different treatments in spleen-derived non-exhausted T cells with/without CD3/CD28 stimulation (n=5). *P < 0.01, ****P < 0.0001 by one-way analysis of variance (ANOVA) with Turkey’s multiple comparisons.


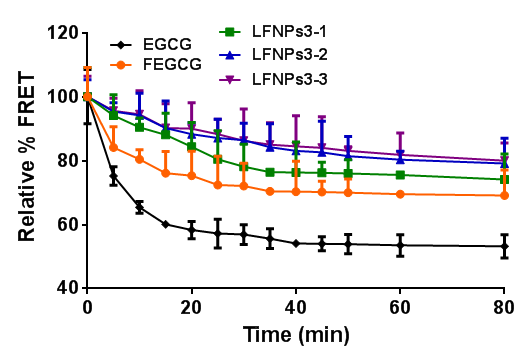


**Figure S7**. Stability of different complexes against disassembly with heparin (n=3).


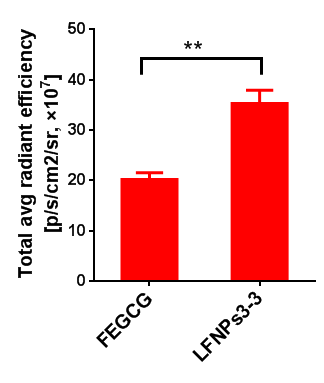


**Figure S8.** Total Cy5-siRNA retention at 12 h postinjection determined from isolated organs (n=3). **P < 0.01 by an unpaired two-tailed Student’s t-test.


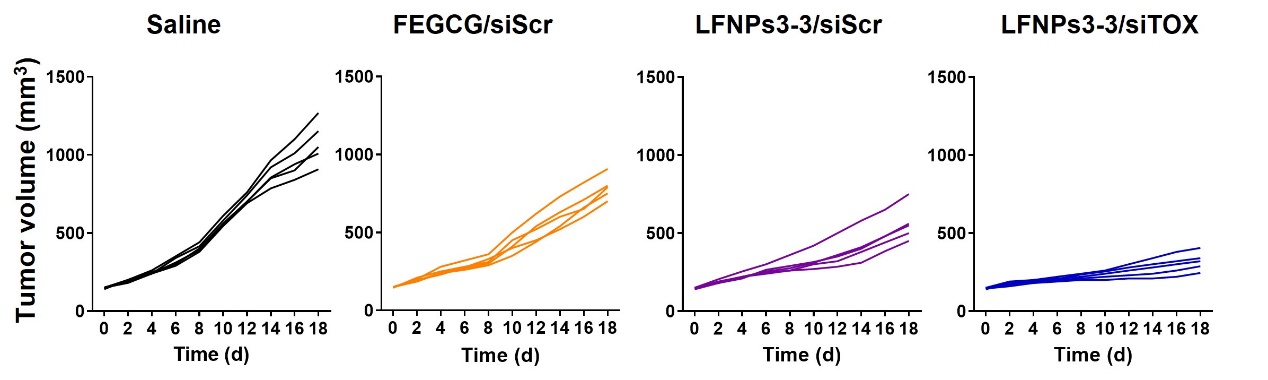


**Figure S9.** The tumor growth curves of individual mouse in different treatments (n=5).


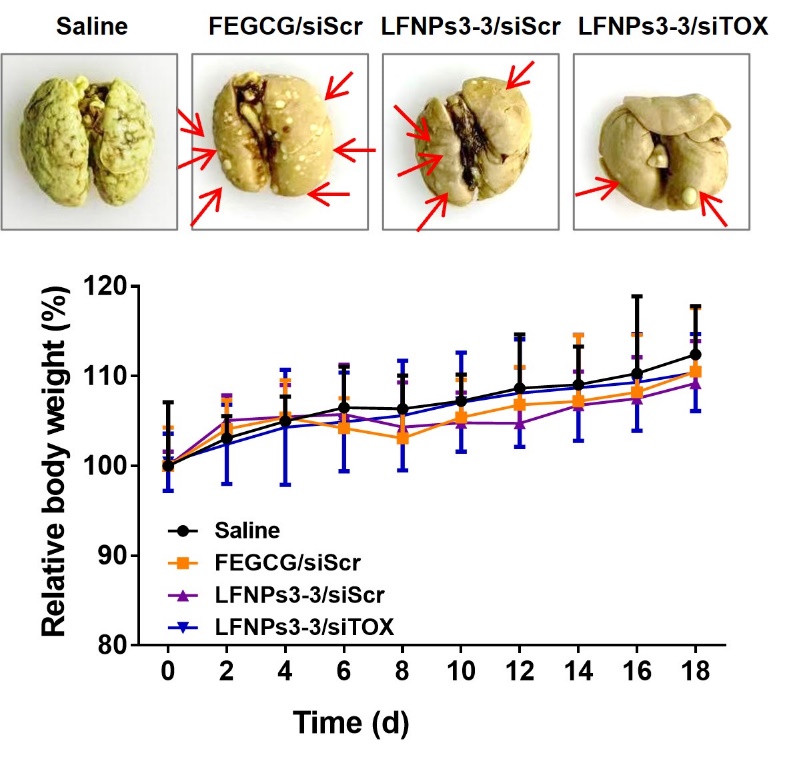


**Figure S10**. Lung image and body weight change (n=5).


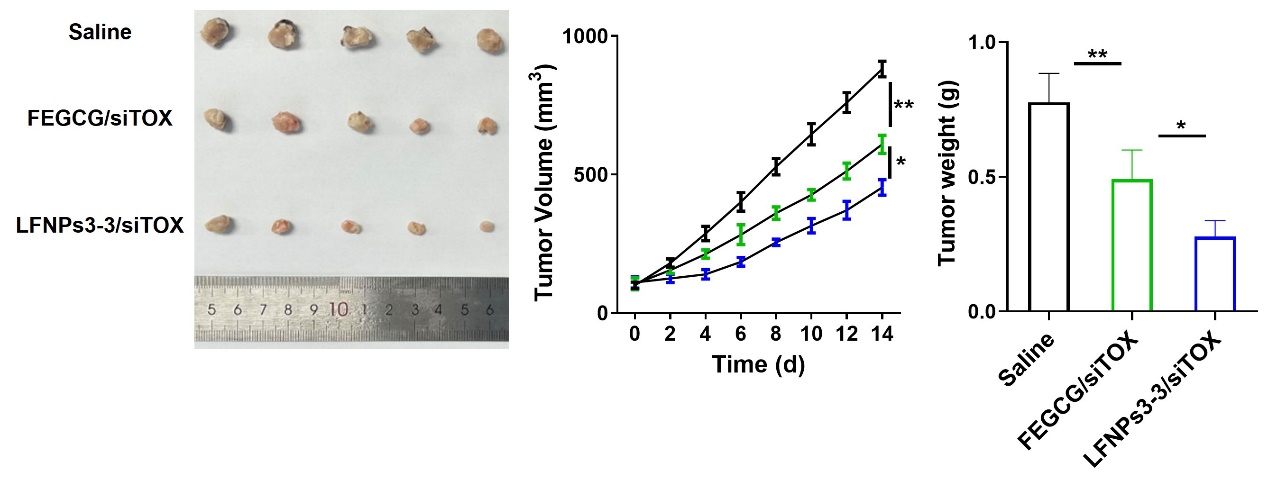


**Figure S11**. Tumor image, tumor volume, and tumor weight after different treatments with saline, FEGCG/siTOX, and LFNPs3-3/siTOX (n=5). *P < 0.05, **P < 0.01 by one-way analysis of variance (ANOVA) with Turkey’s multiple comparisons.


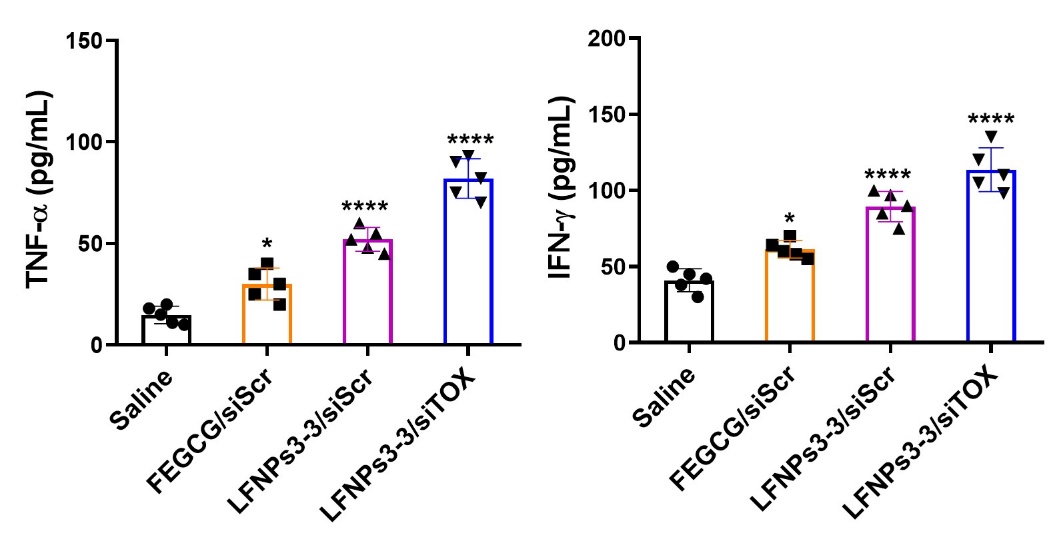


**Figure S12**. TNF-α and IFN-γ levels in plasma after different treatments (n=5). *P < 0.05, ****P<0.01 by one-way analysis of variance (ANOVA) with Turkey’s multiple comparisons.


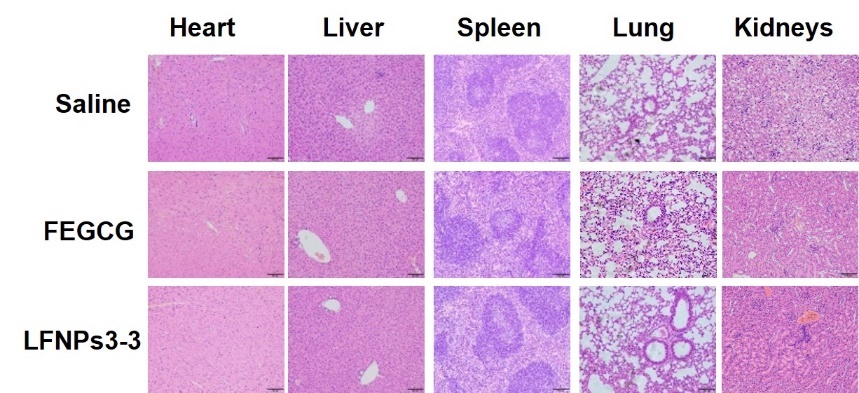


**Figure S13**. H&E staining of major organ tissues in healthy mice.


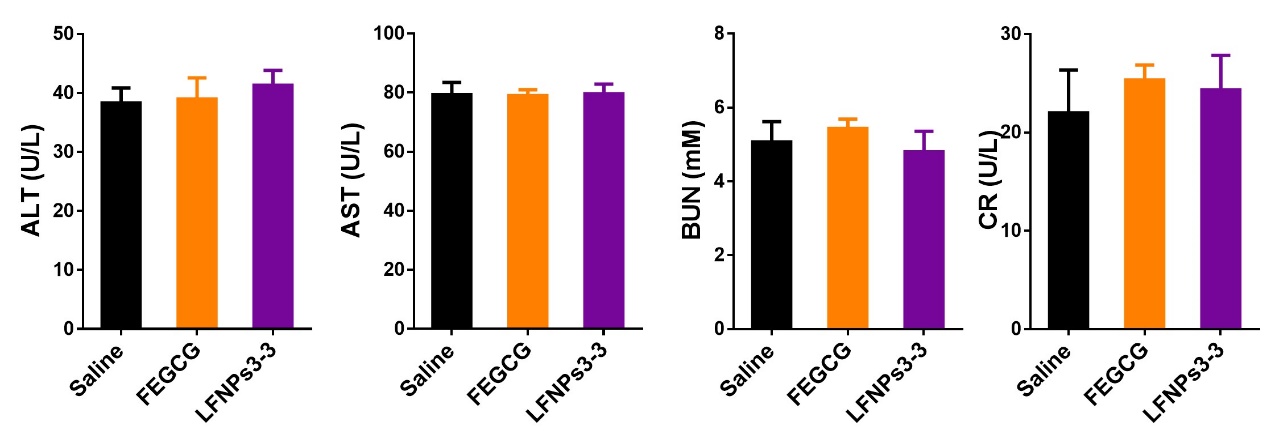


**Figure S14**. Serum biomarkers levels of ALT, AST, BUN and CR (n=5).


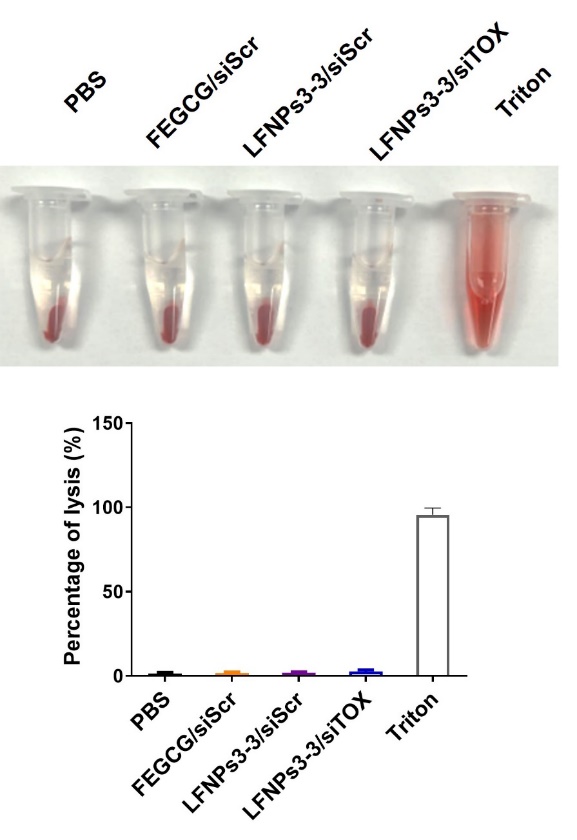


**Figure S15**. Hemolysis rate of FEGCG/siScr, LFNPs3-3/siScr, and LFNPs3-3/siTOX complexes with Triton as positive control in red blood cells (n=5).
